# Evidence and potential mechanism of action of indigo naturalis and its active components in the treatment of psoriasis

**DOI:** 10.1080/07853890.2024.2329261

**Published:** 2024-09-24

**Authors:** Chunxiao Wang, Panpan Yang, Jiao Wang, Liu Liu, Jiale Chen, Xiaoce Cai, Miao Zhang, Naixuan Lin, Sihan Wang, Yuanting Yu, Linge Li, Xin Li

**Affiliations:** a Department of Dermatology, Yueyang Hospital of Integrated Traditional Chinese and Western Medicine, Shanghai University of Traditional Chinese Medicine, Shanghai, China; b Institute of Dermatology, Shanghai Academy of Traditional Chinese Medicine, Shanghai, China; c Department of Dermatology, Shijiazhuang TCM Hospital, Shijiazhuang, China

**Keywords:** Psoriasis, indigo naturalis, efficacy, systematic review, cell, *Strobilanthes cusia* (nees) kuntze

## Abstract

**Background:**

Indigo naturalis is effective against psoriasis. Indigo, indirubin and tryptanthrin, the main active components of indigo naturalis, have anti-inflammatory properties.

**Objective:**

To evaluate the efficacy and safety of indigo naturalis and its active components in the treatment of psoriasis.

**Methods:**

Seven databases were searched for studies of indigo naturalis and its active components for the treatment of psoriasis.

**Results:**

The findings demonstrated a higher response rate in the Chinese herbal medicine (CHM) formula groups than in the control group for Psoriasis Area and Severity Index 60 (PASI60) (Rate difference [RD] = 0.22, *p* < .0001). Among all adverse events, only the incidence of gastrointestinal adverse reactions was higher in the CHM formula group than in the control group (RD = 0.09, *p* < .0001). In preclinical *in vivo* studies, indirubin showed better performance in improving the phenotype of psoriasis-like mice compared to that in controls, including the PASI score (mean difference [MD] = −3.58, *p* < .0001), epidermal thickness (MD = −29.13, *p* < .0001), interleukin-(IL) 17 A mRNA expression (MD = −2.27, *p* = .0066) and IL-23 mRNA (MD = −5.36, *p* = .01).

**Conclusion:**

Indigo naturalis combined with conventional treatments is useful for treating psoriasis. Indigo naturalis display anti-proliferative, anti-inflammatory and anti-angiogenic effects by regulating the TAK1, JAK3/STAT3, Wnt/β-catenin, Akt/PKB, FAK and AP-1/c-Jun pathway.

## KEY MESSAGES

1. Indigo naturalis, a Chinese herb and its main active components, indigo, indirubin and tryptanthrin are effective in treating psoriasis.
2. Indigo naturalis, indirubin, indigo and tryptanthrin have anti-proliferative, anti-inflammatory and anti-angiogenic effects via regulating the TAK1, JAK3/STAT3, Wnt/β-catenin, Akt/PKB, FAK and AP-1/c-Jun pathways.

## 1. Introduction

Psoriasis is a chronic inflammatory skin disease that affects approximately 3.6% of Caucasians [1]. In 2013, psoriasis treatment in the United States accounted for approximately $35.2 billion [2]. Currently, psoriasis is incurable with interleukin-17A (IL-17A) and IL-23 has been identified as a key driver of its progression [3]. Immune-targeted biological therapies can significantly improve the quality of life of patients with moderate-to-severe psoriasis [4]. However, these have side effects [5] and incur high economic costs. Therefore, effective alternative therapies need to be developed.

*Strobilanthes cusia* (Nees) Kuntze, a traditional Chinese medicinal plant, has been widely used for the past 1000 years as an antipyretic-alexipharmic drug. Indigo naturalis (IN, Qingdai) is a processed product of the leaves and stems. All the main active components of IN, including indigo, indirubin (IR) and tryptanthrin, have anti-inflammatory effects and regulate the genetic expression of inflammation-related cytokines [6–8].

Although IN is effective against psoriasis, no systematic reviews have been conducted on its therapeutic efficacy or safety. This study aimed to conduct a systematic review and meta-analysis of IN and its active components in the treatment of psoriasis and to summarize their mechanisms of action.

## 2. Methods and analyses

### 2.1. Search strategy

Databases such as PubMed, Embase, Cochrane Central Register of Controlled Trials, China National Knowledge Infrastructure, WanFang Database for Chinese Technical Periodicals, VIP Database and SinoMed were searched from 1 January 1970 (inception) to 5 July 2022, for literature on the use of IN and its active components in the treatment of psoriasis. In addition, an article by Nguyen et al. [9], although not available in the above databases, was manually searched for inclusion, owing to its high relevance to the current study and its scientific research methodology.

We combined the medical subject headings and free text words to retrieve all 49 relevant studies. The following keywords were used: ‘Psoriasis’, ‘Psoriases’, ‘Pustulosis of Palms and Soles’, ‘Pustulosis Palmaris et Plantaris’, ‘Palmoplantaris Pustulosis’, ‘Pustular Psoriasis of Palms and Soles’, ‘Indigo Naturalis’, ‘Qingdai compound’, ‘Qingdaisan’, ‘qing dai’, ‘Indigo Naturalis ointment’, ‘Indigo’, ‘Indigo Carmine’, ‘Carmine, Indigo’, ‘Soluble Indigo Blue’, ‘Indigo Blue, Soluble’, ‘Indigotindisulfonate Sodium’, ‘2-(1,3-Dihydro-3-oxo-5-sulpho-2H-indol-2-ylidene)-3- oxoindoline-5-sulphonic acid’, ‘Indigotindisulfonic Acid’, ‘Indigotindisulfonate’, ‘Indigo Disulfonate’, ‘FD and C Blue No. 2’, ‘D and C Blue NO. 6’, ‘Indigo Blue’, ‘Indigotin’, ‘(delta-2,2′-biindole)-3,3′-dione’, ‘indirubin’, ‘indigo red’, ‘tryptanthrine’, ‘indolo(2,1-b)quinazolin-6,12-dione’, ‘6,12-dihydro-6,12-dioxoindole(2,1-b)quinazoline’, ‘indolo (2, 1b) quinazoline-6, 12 dione’, and ‘tryptanthrin.’

### 2.2. Inclusion criteria

Clinical studies: (1) randomized clinical trials (RCTs), (2) the Psoriasis Area and Severity Index (PASI) score were used as outcome indicators and (3) patients with definite diagnostic standards of psoriasis were included.

Preclinical studies: psoriasis-like mouse models with control comparisons or psoriasis-like cell models with control comparisons.

The exclusion criteria were as follows: (1) case reports, reviews, conferences, repeat studies and scientific or technological achievements, (2) unreasonable setting of the control group.

### 2.3. Data extraction

Two authors (C-X W and P-P Y) independently screened the literature and extracted data.

Clinical studies: first author, year of publication, sample size, mean or median age of the participants, sex, duration of psoriasis, characteristics of intervention and control groups and information on efficacy and adverse events.

Preclinical studies: First author, year of publication, sample size, characteristics of the animals, including species, sex, weight, intervention characteristics and result indicators.

### 2.4. Risk of bias assessment

The Cochrane Collaboration tool was used to assess the risk of bias in clinical trials. The Systematic Review Center for Laboratory Animal Experimentation (SYRCLE) was used to assess the risk of bias in preclinical trials. Three reviewers (N-X L, S-H W and Y-T Y) independently assessed the quality of clinical and preclinical studies. If a disagreement presented in the evaluation, the researcher (LL) participated in the discussion to reach a consensus.

### 2.5. Statistical analyses

R-4.1.3 was used for data analyses. Q-text and I^2^ statistics were used to test heterogeneity of the data, and the random-effects model was used if I^2^ > 50%. Rate differences (RD) with 95% confidence intervals (CI) were calculated for dichotomous data and mean differences (MD) with 95% CI were calculated for continuous variables.

## 3. Results

### 3.1. Selection of studies

A total of 490 articles were initially retrieved by searching subject headings and free words. Of these, 293 duplicates and irrelevant studies were removed, while 50 case reports, letters, conference papers, commentaries, and reviews were excluded after reading the abstracts and full text. Seventy-five articles were excluded because they were not RCTs, did not include an appropriate outcome, or lacked appropriate comparisons. Five articles had incomplete data. Eighteen articles had no full texts. Finally, 49 articles were included in our meta-analyses, including 26 RCTs and 23 preclinical studies (Supplementary Figure S1).

### 3.2. Characteristics of included studies

The 26 clinical trials comprised of six interventions in form of decoctions containing IN and Chinese herbal medicine (CHM) formulas with IN as the major herb (IND), topical CHM formulas, such as ointments and oils containing IN (INT), other oral drugs (OOD) including diyin tablets, acitretin, compound aminopeptide tablets, methotrexate and compound glycyrrhizin capsule, light therapy (LT), and other topical therapies (OTT) such as calcipotriol ointment, tacrolimus ointment and placebo (PBO). We selected PASI60 as the primary efficacy outcome. Three studies [10–12] were used to compare the efficacies of IND and OOD. Five studies [13–17] were used to compare the combined use of IND and OOD against OOD alone. Four studies [18–21] compared the combined use of IND and LT against LT alone. Five studies [22–26] compared the combined use of IND, OOD and OTT with that of OOD and OTT alone. One study [27] compared the combined use of INT and OOD with that of OOD alone. One study [28] compared use of INT against placebo. A study [29] was used to compare the efficacy of INT to that of OTT (Supplementary Table S1). Of the total preclinical studies used, seven were *in vivo* [30–36] and 15 were *in vitro* studies [6,30,34,36–47]. Five studies [31,33–36] used BALB/c mice, one [30] used IKK2 knockout mice, while another [32] used Hartley guinea pigs. The routes of administration included subcutaneous injection [35,36], gastric filling [33,34] and topical use [30–32]. The dosage forms of IN for external use included oil [32], ointment [30] and micro emulsification (ME) gel [31]. Five studies used an imiquimod-induced (IMQ-induced) psoriasis-like mouse model [31,33–36], while one employed a model of psoriatic lesions induced by propranolol hydrochloride [32]. One study used male mice bearing a tamoxifen-induced (TMF-induced) IKK2 deletion in the epidermis [30].

Five cells lines employed in the systematic review of the vitro experiments included human keratinocytes [30,40,43,44], human immortal keratinocyte (HaCaT) cells [34,36,42,45,47], γδ T cells [34], CD4+ T cells [6,41] and human umbilical vein endothelial cells [37–39]. We prepared a checklist of IN and its main components as well as *in vivo* and *in vitro* studies that included access, route of administration, experimental concentration, dose and preparation method **(**Supplementary Table S2).

### 3.3. Risk of bias

#### 3.3.1. Clinical studies

Supplementary Figures S2 and S3 show the risks of bias in the 26 studies that were included. Of these, six studies [13,17,21,22,25,48] used the random number table method, while 20 studies [10–12,14–16,18–20,23,24,26–29,49–53] used the random method. Allocation concealment was employed by only one study [48]. Six studies [10,12,15,19,27,29] failed to mention blinding. All studies reported complete results.

#### 3.3.2. Preclinical studies

The SYRCLE tool was used to evaluate the risk of bias in the eight preclinical animal experiments. One study [36] used the random number table method, and three studies [32,34] used the random method. Three studies [32,34,36] provided the baseline data. None of the studies used employed allocation concealment or blinding, suggesting a high risk of bias. One study [36] mentioned a standard diet while another [34] mentioned single-cage feeding. None of the studies mentioned whether blind method was used for animal selection and outcome evaluation. However, all the studies reported bias in the data such as, incomplete outcome data, selective outcome reporting and other sources of bias. Above mentioned factors thus led to the overall quality of the included studies to be relatively low (Supplementary Figure S4).

### 3.4. Primary outcomes of the clinical studies

#### 3.4.1. PASI60

PASI 60 refers to a 60% reduction in the PASI score of patients and is considered an indicator of the effectiveness of psoriasis treatment. A meta-analyses of 20 clinical trials [10–29] evaluated PASI60 in patients and divided them into seven subgroups. Combined with IN can increase the efficacy of psoriasis treatment (RD = 0.22, 95% CI [0.16; 0.28], *p* < .0001), IND + OOD and OOD (RD = 0.25, 95% CI [0.16; 0.34], *p* < .0001, I^2^ = 0%), IND+LT and LT (RD = 0.16, 95% CI [0.07; 0.25], *p* < .0005, I^2^ = 0%), IND+OOD+OTT and OOD+OTT (RD = 0.19, 95% CI [0.11; 0.27], *p* < .0001, I^2^ = 0%), INT+OOD and OOD (RD = 0.23, 95% CI [0.07; 0.39], *p* < .0001), INT and PBO (RD = 0.58, 95% CI [0.42; 0.74], *p* < .0001). However, the statistical differences between IND and OOD (*p* = .1556) and INT and OTT (*p* = .0651) were insignificant, indicating that IN monotherapy, whether systemic or topical, had no difference as compared to that in the control groups. The details have been presented in Table 1.

**Table 1.** Subgroup analysis of PASI60 in clinical studies.

Study	Comparison	Experimental group		Control group		Risk difference	*p* Value
		Events	Total	Events	Total	[95% CI]	
1. PASI60							
1.1 IND VS. OOD							
Chen et al. [10]	IND VS. OOD	33	61	32	55	−0.04 [−0.22; 0.14]	–
Liang et al. [11]	IND VS. OOD	23	30	15	30	0.27 [ 0.03; 0.50]	–
Wang et al. [12]	IND VS. OOD	24	30	16	30	0.27 [ 0.04; 0.50]	–
Subtotal (95% CI) I^2^ = 67%						0.15 [−0.06; 0.36]	.1556
1.2 IND + OOD VS. OOD							
Xue et al. [16]	IND + OOD VS. OOD	28	34	19	34	0.26 [ 0.05; 0.48]	–
Cai et al. [13]	IND + OOD VS. OOD	22	33	10	33	0.36 0.14; 0.59]	–
Wu et al. [15]	IND + OOD VS. OOD	45	60	21	50	0.33 [ 0.15; 0.51]	–
Zhang et al. [17]	IND + OOD VS. OOD	46	60	38	60	0.13 [−0.03; 0.30]	–
Pang et al. [14]	IND + OOD VS. OOD	35	45	25	45	0.22 [ 0.03; 0.41]	–
Subtotal (95% CI) I^2^ = 0%						0.25 [ 0.16; 0.34]	<.0001
1.3 IND + LT VS. LT							
Xie et al. [20]	IND + LT VS. LT	31	49	24	48	0.13 [−0.06; 0.33]	–
Yang et al. [21]	IND + LT VS. LT	37	58	28	58	0.16 [−0.02; 0.33]	–
Ji [18]	IND + LT VS. LT	35	52	25	52	0.19 [ 0.01; 0.38]	–
Wang [19]	IND + LT VS. LT	37	44	29	43	0.17 [−0.01; 0.34]	–
Subtotal (95% CI) I^2^ = 0%						0.16 [ 0.07; 0.25]	.0005
1.4 IND + OOD + OTT VS. OOD + OTT							
Han et al. [22]	IND + OOD + OTT VS. OOD + OTT	41	51	30	50	0.2 [ 0.03; 0.38]	–
Yang et al. [21]	IND + OOD + OTT VS. OOD + OTT	25	41	20	41	0.12 [-0.09; 0.34]	–
Lin [25]	IND + OOD + OTT VS. OOD + OTT	45	50	34	50	0.22 [ 0.07; 0.37]	–
Lin et al. [24]	IND + OOD + OTT VS. OOD + OTT	29	31	23	31	0.19 [ 0.02; 0.37]	–
Han et al. [23]	IND + OOD + OTT VS. OOD + OTT	42	48	33	48	0.19 [ 0.03; 0.35]	–
Subtotal (95% CI) I^2^ = 0%						0.19 [ 0.11; 0.27]	<.0001
1.5 INT + OOD VS. OOD							
Chen [29]	INT + OOD VS. OOD	50	60	34	56	0.23 [ 0.07; 0.39]	–
Subtotal (95% CI)						0.23 [ 0.07; 0.39]	.0053
1.6 INT VS. PBO							
Lin et al. [28]	INT VS. PBO	36	48	8	48	0.58 [ 0.42; 0.74]	–
Subtotal (95% CI)						0.58 [ 0.42; 0.74]	<.0001
1.7 INT VS. OTT							
Chen et al. [29]	INT VS. OTT	17	20	12	20	0.25 [−0.02; 0.52]	–
Subtotal (95% CI)						0.25 [−0.02; 0.52]	.0651
Total (95% CI) I^2^ =46%						0.22 [0.16; 0.28]	<.0001

*Note:* PASI60: Psoriasis Area and Severity Index 60.

### 3.5. Secondary outcomes of clinical studies

#### 3.5.1. PASI

The PASI score, a quantitative tool for evaluating the severity and extent of psoriasis, is calculated by erythema, induration and desquamation and then multiplied by the area affected. We evaluated the PASI scores of patients with psoriasis from 13 studies [11,12,14,17–19,21,23,24,27,28,49,52,53] and concluded that the efficacy of IND was better than that of OOD (mean difference [MD] = −2.31, 95%CI [−3.79; −0.83], *p* = .0022, I^2^ = 0%), the effect of IND combined with OOD was better than that of OOD alone (MD = −2.78, 95%CI [−3.65; −1.91], *p* < .0001, I^2^ = 0%), that of IND + LT was better than LT alone (MD = −3.54, 95%CI [−3.70; −3.37], *p* < .0001, I^2^ = 50%), IND + OOD + OTT was better than OOD + OTT (MD = −2.41, 95%CI [−3.22; −1.60], *p* < .0001, I^2^ = 0%) and of INT + OOD was better than OOD alone (MD = −4.38, 95%CI [−6.40; −2.36], *p* < .0001). In addition, IND and INT were more effective than placebo (*p* < .0001). However, one study [53] compared INT with betamethasone and concluded a lower clinical response of the prior in comparison with topical betamethasone (MD = 2.99, 95%CI [1.35; 4.62], *p* = .0003, I^2^ = 0%) (Table 2).

**Table 2.** Subgroup analysis of PASI, PSI and shNAPSI in clinical studies.

Study	Comparison	Outcomes (mean ± SD)		Mean difference	*p* Value
		Experiment	Control	[95%CI]	
2. 1 PASI					
2.1.1 IND VS. OOD					
Liang et al. [11]	IND VS. OOD	3.14 ± 3.57	5.45 ± 4.53	−2.31 [−4.37; −0.25]	–
Wang et al. [12]	IND VS. OOD	3.15 ± 3.67	5.46 ± 4.63	−2.31 [−4.42; −0.20]	–
Subtotal (95% CI) I^2^ = 0%				−2.31 [−3.79; −0.83]	.0022
2.1.2 IND + OOD VS. OOD					
Zhang et al. [17]	IND + OOD VS. OOD	16.58 ± 4.90	19.47 ± 4.85	−2.89 [−4.63; −1.15]	–
Pang et al. [14]	IND + OOD VS. OOD	3.81 ± 2.42	6.55 ± 2.43	−2.74 [–3.74; −1.74]	–
Subtotal (95% CI) I^2^ = 0%				−2.78 [–3.65; −1.91]	<.0001
2.1.3 IND + LT VS. LT					
Yang et al. [21]	IND + LT VS. LT	3.11 ± 0.52	6.78 ± 1.01	−3.67 [–3.96; −3.38]	–
Ji [18]	IND + LT VS. LT	5.20 ± 0.50	8.70 ± 0.60	−3.50 [−3.71; −3.29]	–
Wang [19]	IND + LT VS. LT	4.45 ± 2.83	6.98 ± 2.51	−2.53 [−3.65; −1.41]	–
Subtotal (95% CI) I^2^ = 50%				−3.54 [−3.70; −3.37]	<.0001
2.1.4 IND + OOD + OTT VS. OOD + OTT					
Lin et al. [24]	IND + OOD + OTT VS. OOD + OTT	3.03 ± 1.97	5.57 ± 1.93	−2.54 [−3.51; −1.57]	–
Han et al. [23]	IND + OOD + OTT VS. OOD + OTT	3.14 ± 2.15	5.25 ± 4.79	−2.11 [−3.60; −0.62]	–
Subtotal (95% CI) I^2^ = 0%				−2.41 [−3.22; −1.60]	<.0001
2.1.5 INT VS. OTT					
Yazdanpanah et al. [53]	INT VS. OTT	5.80 ± 3.13	3.09 ± 2.23	2.71 [0.33; 5.09]	–
Yazdanpanah et al. [53]	INT VS. OTT	6.32 ± 2.85	3.09 ± 2.23	3.23 [0.99; 5.47]	–
Subtotal (95% CI) I^2^ = 0%				2.99 [1.35; 4.62]	.0003
2.1.6 IND VS. PBO					
Wang [52]	IND VS. PBO	2.12 ± 0.07	9.64 ± 2.46	−7.52 [−8.45; −6.59]	–
Subtotal (95% CI)				−7.52 [−8.45; −6.59]	<.0001
2.1.7 INT VS. PBO					
Cheng et al. [49]	INT VS. PBO	2.64 ± 1.50	8.30 ± 4.00	−5.66 [−8.53; −2.79]	–
Subtotal (95% CI)				−5.66 [−8.53; −2.79]	.0001
2.1.8 INT + OOD VS. OOD					
Chen [27]	INT + OOD VS. OOD	16.18 ± 4.62	20.56 ± 6.28	−4.38 [−6.40; −2.36]	–
Subtotal (95% CI)				−4.38 [−6.40; −2.36]	<.0001
Total (95% CI) I^2^ = 91.4%				−2.66 [−4.05; −1.26]	.0002
2.2 PSI					
Lin et al. [28]	INT VS. PBO	2.27 ± 2.68	5.98 ± 2.58	−3.71 [−4.76, −2.66]	–
Lin et al. [50]	INT VS. PBO	6.43 ± 1.35	12.85 ± 1.41	−6.42 [−7.08, −5.76]	–
Total (95% CI) I^2^ = 95%				−5.10 [−7.75, −2.44]	.0002
2.3 shNAPSI					
** **Lin et al. [48]	INT VS. PBO	10.70 ± 6.20	15.50 ± 6.20	−4.80 [−7.94, −1.66]	–
** **Lin et al. [51]	INT VS. OTT	14.40 ± 8.90	20.40 ± 8.50	−6.00 [−10.20, −1.80]	–
Total (95% CI) I^2^ = 0%				−5.23 [−7.74, −2.72]	<.0001

*Note:* PASI: Psoriasis Area and Severity Index; PSI: psoriasis severity index; shNAPSI: single hand nail psoriasis severity index.

#### 3.5.2. PSI

The Psoriasis Severity Index (PSI) score is a sum of the grading for erythema, induration and scaling on a five-point scale (0 = absent, 1 = slight, 2 = moderate; 3 = severe and 4 = very severe), with the maximum score being 12. Meta-analyses of two studies [28,50] evaluated PSI in patients with recalcitrant plaque-type psoriasis, who topically applied either IN or vehicle ointments. The results showed a significant reduction in the PSI scores (MD = −5.10, 95%CI [−7.75, −2.44], *p* = .0002, I^2^ = 95%), the details for which have been presented in Table 2.

#### 3.5.3. shNAPSI

The Nail Psoriasis Severity Index (NAPSI), the only validated clinical scale, is a simple and objective tool for the clinical assessment of nail psoriasis. The efficacy of INT was superior to that of placebo or OTT (MD = −5.23, 95% CI [−7.74, −2.72], *p* < .001, I^2^ = 0%) (Table 2).

#### 3.5.4. Cytokines and cells

Six clinical studies have described changes in cytokines and immune cells in the serum of patients with psoriasis after IND treatment. The number of CD4^+^ T cells increased (MD = 4.84, 95% CI [3.6143; 6.07], *p* < .0001) while that of CD8^+^ T cells decreased (MD = −4.11, 95% CI [−5.07; −3.15], *p* < .0001) in the serum of patients compared to serum from control participants, thus increasing the CD4^+^ T cell/CD8^+^ T cell ratio (MD = 0.41, 95% CI [−0.30; 0.52], *p* < .0001). Compared to the control group, patients treated with IND had increased serum IL-4 levels (MD = 5.39, 95% CI [4.55; 6.23], *p* < .0001, I^2^ = 0%) and decreased serum IL-8 levels (MD = −4.88, 95% CI [−7.34; −2.41], *p* = .0001, I^2^ = 0%). Additionally, IND treatment decreased TNF-α levels (MD = −12.83, 95% CI [−16.76; −8.90], *p* < .0001, I^2^ = 0%) and IL-17 (MD = −14.28, 95% CI [−18.30; −10.26], *p* < .0001, I^2^ = 41%) in the serum of patients compared to untreated control serum samples; however, no statistically significant change in IL-23 levels was observed compared to the control group (MD = −44.18, 95% CI [−89.23; 0.87], *p* = .0546, I^2^ = 98%). Details are presented in Table 3.

**Table 3.** Analysis of cells and cytokines in clinical studies.

Study	Comparison	Outcomes (mean ± SD)		Mean difference	*p* Value
		Experiment	Control	[95%CI]	
3.1 CD4+ T cell					
Zhang et al. [17]	IND + OOD VS. OOD	41.92 ± 3.46	37.08 ± 3.39	4.84 [3.6143; 6.07]	<.0001
3.2 CD8+ T cell					
Zhang et al. [17]	IND + OOD VS. OOD	24.40 ± 2.65	28.51 ± 2.72	−4.11 [−5.07; −3.15]	<.0001
3.3 CD4 T + cell /CD8+ T cell					
Zhang et al. [10]	IND + OOD VS. OOD	1.72 ± 0.33	1.31 ± 0.30	0.41 [0.30; 0.52]	<.0001
3.4 IL-4					
Pang et al. [14]	IND + OOD VS. OOD	22.89 ± 3.15	17.22 ± 2.83	5.67 [4.43; 6.91]	
Lin [25]	IND + OOD + OTT VS. OOD + OTT	23.01 ± 3.16	17.86 ± 2.65	5.15 [4.01; 6.29]	
Total (95% CI) I^2^ = 0%				5.39 [4.55; 6.23]	<.0001
3.5 IL-8					
Pang et al. [14]	IND + OOD VS. OOD	45.81 ± 9.63	50.33 ± 9.41	−4.52 [−8.45; −0.59]	
Lin [25]	IND + OOD + OTT VS. OOD + OTT	46.14 ± 7.92	51.25 ± 8.23	−5.11 [−8.28; −1.94]	
Total (95% CI) I^2^ = 0%				−4.88 [−7.34; −2.41]	.0001
3.6 TNF-α					
Cai et al. [13]	IND + OOD VS. OOD	95.20 ± 17.10	109.20 ± 16.20	−14.00 [−22.04; −5.96]	
Yang et al. [21]	IND + LT VS. LT	93.26 ± 11.79	105.43 ± 13.97	−12.17 [−17.77; −6.57]	
Xie et al. [20]	IND + LT VS. LT	94.20 ± 17.40	107.20 ± 17.60	−13.00 [−20.58; −5.42]	
Total (95% CI) I^2^ = 0%				−12.83 [−16.76; −8.90]	<.0001
3.7 IL-17					
Cai et al. [13]	IND + OOD VS. OOD	161.20 ± 17.10	182.20 ± 17.80	−21.00 [−29.42; −12.58]	
Yang et al. [21]	IND + LT VS. LT	95.23 ± 10.14	106.72 ± 15.48	−11.49 [−17.15; −5.83]	
Xie et al. [20]	IND + LT VS. LT	166.80 ± 17.80	180.60 ± 18.10	−13.80 [−21.57; −6.03]	
Total (95% CI) I^2^ = 41%				−14.28 [−18.30; −10.26]	<.0001
3.8 IL-23					
Cai et al. [13]	IND + OOD VS. OOD	203.20 ± 15.80	293.60 ± 28.90	−90.40 [−101.64; −79.16]	
Yang et al. [21]	IND + LT VS. LT	187.25 ± 13.49	209.78 ± 16.42	−22.53 [ −29.03; −16.03]	
Xie et al. [20]	IND + LT VS. LT	192.50 ± 22.70	212.50 ± 24.60	−20.00 [ −30.25; −9.75]	
Total (95% CI) I^2^ = 98%				−44.18 [−89.23; 0.87]	.0546

#### 3.5.5. Adverse events

Eighteen articles reported adverse events associated with psoriasis treatment, with the most common being gastrointestinal reactions, skin dryness, itching, dry mouth and erythema. These articles were divided into two groups: the external drug group (Supplementary Figure S5) and the systematic drug group (Supplementary Figure S6). Increased gastrointestinal reactions were reported in the systemic drug group compared to the control group (RD = 0.09, 95% CI [0.05; 0.13], *p* < .0001). However, other side effects in the systematic drug group and all side effects in the external drug group showed no statistically significant differences compared with the control group (Supplementary Figure S5 and S6).

### 3.6. Primary outcomes of preclinical *in vivo* studies

#### 3.6.1. Psoriasis area and severity index of IR for psoriasis

One study [34] assessed the total scores in an IR-treated psoriasis mouse model on day 7, IR significantly reduced the total scores compared to those of the controls (MD = −3.58, *p* < .0001, 95% CI [−4.11; −3.05]). At the same time, the scale (MD = −1.08, *p* < .0001, 95% CI [−1.26; −0.91]), thickness (MD = −1.19, *p* < .0001, 95% CI [−1.30; −1.07]) and erythema (MD = −1.31, *p* < .0001, 95% CI [−1.54; −1.08]) scores displayed improvement compared with those of the controls (Table 4).

**Table 4.** Analysis of PASI scores and epidermal thickness *in vivo* preclinical studies.

Study	Outcomes (mean ± SD)		Mean difference	*p* Value
	Experiment	Control	[95%CI]	
4.1 Total PASI sore				
Xie et al. [33] − 1	2.84 ± 0.60	6.59 ± 0.86	−3.75 [−4.59; −2.91]	–
Xie et al. [33] − 2	2.99 ± 0.68	6.59 ± 0.86	−3.60 [−4.48; −2.72]	–
Xie et al. [33] − 3	3.34 ± 1.07	6.59 ± 0.86	−3.25 [−4.35; −2.15]	–
Total (95% CI) I^2^ = 0%			−3.58 [−4.11; −3.05]	<.0001
4.2 Scales				
Xie et al. [33] − 1	0.92 ± 0.24	2.06 ± 6.00	−1.14 [−1.43; −0.85]	–
Xie et al. [33] − 2	1.02 ± 0.20	2.06 ± 6.00	−1.04 [−1.31; −0.77]	–
Xie et al. [33] − 3	0.99 ± 0.42	2.06 ± 6.00	−1.07 [−1.47; −0.67]	–
Total (95% CI) I^2^ = 0%			−1.08 [−1.26; −0.91]	<.0001
4.3 Thickness				
Xie et al. [33] − 1	1.08 ± 0.14	2.32 ± 0.21	−1.24 [−1.44; −1.04]	–
Xie et al. [33] − 2	1.08 ± 0.08	2.32 ± 0.21	−1.24 [−1.42; −1.06]	–
Xie et al. [33] − 3	1.29 ± 0.20	2.32 ± 0.21	−1.03 [−1.26; −0.80]	–
Total (95% CI) I^2^ = 15%			−1.19 [−1.30; −1.07]	<.0001
4.4 Erythema				
Xie et al. [33] − 1	0.83 ± 0.78	2.25 ± 0.34	−1.42 [−2.10; −0.73]	–
Xie et al. [33] − 2	0.89 ± 0.19	2.25 ± 0.34	−1.36 [−1.67; −1.05]	–
Xie et al. [33] − 3	1.06 ± 0.39	2.25 ± 0.34	−1.19 [−1.60; −0.78]	–
Total (95% CI) I^2^ = 0%			−1.31 [−1.54; −1.08]	<.0001
4.5 Epidermal thickness				
Xie et al. [33] − 1	36.10 ± 7.32	70.52 ± 5.69	−34.42 [−41.84; −27.01]	–
Xie et al. [33] − 2	41.65 ± 4.88	70.52 ± 5.69	−28.87 [−34.87; −22.87]	–
Xie et al. [33] − 3	45.58 ± 5.69	70.52 ± 5.69	−24.94 [−31.38; −18.50]	–
Total (95% CI) I^2^ = 44%			−29.13 [−34.18; −24.08]	<.0001

*Note:* PASI: Psoriasis Area and Severity Index.

#### 3.6.2. Epidermal thickness

Compared to that of the controls, epidermal thickness in psoriasis-like mice was significantly reduced seven days after the IR treatment (MD = −29.13, *p* < .0001, 95% CI [−34.18; −24.08]) (Table 4).

In a similar comparison, another meta-analyses from a study [9] clarified that IL-22-induced mouse ear thickness was reduced by topical IR treatment (MD = −0.05, *p* < .0001, 95% CI [−0.07; −0.03]) (Supplementary Table S4).

#### 3.6.3. Cytokine gene expression

Effects of the inflammatory mediators, post the IR treatment, in psoriasis-like mice were assessed by two studies [34,36]. IR significantly reduced the mRNA expression of IL-17A (MD = −2.27, *p* = .0066, 95% CI [−3.92; −0.63]) and IL-23 (MD = −5.36 [−13.74; 3.03], *p* = .01, 95% CI [−13.74; 3.03]) in psoriasis-like mice compared to those of the controls. However, no such effects were observed in IL-22 mRNA expression (MD = −18.74, *p* = .0513, 95% CI [−37.60; 0.11]) (Table 5).

**Table 5.** Analysis of cytokines and cytokine genes expression and cells cycle *in vivo* preclinical studies.

Study	Outcomes (mean ± SD)		Mean difference	*p* Value
	Experiment	Control	[95%CI]	
5.1 IL-17A mRNA				
Xie et al. [33]	1.39 ± 0.44	2.12 ± 0.16	−3.18 [−4.13; −2.23]	–
Xue [36]	4.57 ± 0.86	3.62 ± 0.27	−1.50 [−1.72; −1.28]	–
Total (95% CI) I^2^ = 91%			−2.27 [−3.92; −0.63]	.0066
5.2 IL-23 mRNA				
Xie et al. [33]	0.89 ± 0.35	1.98 ± 0.67	−1.09 [ −1.83; −0.35]	–
Xue [36]	7.46 ± 1.26	17.10 ± 0.94	−9.65 [−10.74; −8.56]	–
Total (95% CI) I^2^ = 99%			−5.36 [−13.74; 3.03]	.01
5.3 IL-22 mRNA				
Xie et al. [33]	8.54 ± 5.24	17.32 ± 5.12	−8.78 [−15.96; −1.60]	–
Xue [36]	23.84 ± 0.92	51.87 ± 1.38	−28.03 [−29.18; −26.88]	–
Total (95% CI) I^2^ = 96%			−18.74 [−37.60; 0.11]	.0513
5.4 G0/G1 phase				
Liu et al. [45] − 1	44.39 ± 1.16	33.29 ± 0.92	11.10 [9.42; 12.77]	–
Liu et al. [45] − 2	53.64 ± 1.39	33.29 ± 0.92	20.35 [18.46; 22.23]	–
Liu et al. [45] − 3	63.82 ± 1.85	33.29 ± 0.92	30.52 [28.18; 32.86]	–
Total (95% CI) I^2^ = 99%			20.63 [9.65; 31.62]	.0002
5.5 S phase				
Liu et al. [45] − 1	42.54 ± 1.16	41.85 ± 0.69	0.69 [ −0.83; 2.22]	–
Liu et al. [45] − 2	31.45 ± 0.69	41.85 ± 0.69	−10.40 [−11.51; −9.29]	–
Liu et al. [45] − 3	24.51 ± 1.39	41.85 ± 0.69	−17.34 [−19.10; −15.59]	–
Total (95% CI) I^2^ = 99%			−9.01 [−19.29; 1.27]	.0858
5.6 TNF-α				
Li et al. [42]-1	1.30 ± 0.10	1.55 ± 0.09	−0.25 [−0.40; −0.10]	–
Li et al. [42]-2	1.06 ± 0.08	1.55 ± 0.09	−0.49 [−0.63; −0.35]	–
Li et al. [42]-3	0.90 ± 0.18	1.55 ± 0.09	−0.65 [−0.88; −0.42]	–
Nguyen et al. [9]	14.75 ± 0.64	17.08 ± 0.85	−2.33 [−3.53; −1.13]	–
Total (95% CI) I^2^ = 84%			−0.55 [−0.83; −0.26]	.0002
5.7 IL-6				
Li et al. [42]-1	1.35 ± 0.21	1.68 ± 0.05	−0.33 [ −0.57; −0.09]	–
Li et al. [42]-2	0.50 ± 0.16	1.68 ± 0.05	−1.18 [−1.37; −0.99]	–
Li et al. [42]-3	0.42 ± 0.19	1.68 ± 0.05	−1.26 [−1.48; −1.04]	–
Nguyen et al. [9]	34.74 ± 5.68	53.05 ± 5.47	−18.31 [−27.23; −9.39]	–
Total (95% CI) I^2^ = 94%			−1.01 [−1.65; −0.38]	.002

### 3.7. Primary outcome of preclinical studies *in vitro*

#### 3.7.1. Cell cycle

IR can arrest HaCaT cells in the G0/G1 phase (MD = −20.63, *p* = .0002, 95% CI [9.65; 31.62]); there was however no evidence to indicate any effect of IR on the S phase of the HaCaT cell cycle (MD = −9.01, *p* = .0858, 95% CI [−19.29; 1.27]) (Table 5).

#### 3.7.2. Cytokine

IR could significantly decrease the production of TNF-α (MD = −0.55, *p* = .0002, 95% CI [−0.83; −0.26]) and IL-6 (MD = −1.01, *p* = .002, 95% CI [−1.65; −0.38]) in IL-22-treated or non-IL-22-treated HaCaT cells (Table 5).

## 4. Discussion

### 4.1. Summary of evidence

Our systematic review included 49 studies: 26 clinical trials and 23 preclinical trials. Evidence from 26 clinical trials, involving 1952 participants, showed that decoctions used for psoriasis treatment, containing IN and CHM formulas with IN as the major herb can reduce PASI, PSI and shNAPSI scores. Preclinical studies comprising eight animal experiments on 279 mice and 16 cell experiments involving five cell lines demonstrated that IN, IR, indigo and tryptanthrin had anti-proliferative, anti-inflammatory and anti-angiogenic effects in animal models of psoriasis.

### 4.2. Limitations

Though clinical research has identified the efficacy of CHM formulations, it has not been substantiated enough by the toxicological and pharmacological studies, quality control of each component, product information of the formulas and authentication methods. Owing to the complex composition of CHM formulations, it is difficult to identify therapeutic and harmful ingredients therein. The follow-up periods of these research were relatively short and rarely focused on disease recurrence or long-term adverse effects, rendering the quality of the clinical trials included in these meta-analyses to be low. Moreover, a comparison between the use of IN alone and the standard treatment for psoriasis is lacking. Finally, most of the included cell experiments reported single and incomplete signaling pathways, which made it difficult to confirm the key targets of the mechanisms of psoriatic IN treatment.

### 4.3. Implications

A combination with IN can improve the curative effects of conventional treatments used to alleviate psoriasis. These convention treatment strategies involve the use of methotrexate, amino peptide tablets, calcipotriol cream, mometasone furoate and acitretin. However, the curative effect of a single drug is weaker than that of hormones. These adverse effects make IN a complementary treatment option for psoriasis.

The pathogenesis of psoriasis involves multiple processes. Keratinocytes play an important role in the regulation of skin inflammation through the release of proinflammatory cytokines, chemokines and antimicrobials. Two studies have shown that IN, IR and tryptanthrin can reduce the expression of anti-microbial S100A9 peptide, chemokine (C–C motif) ligand 20 (CCL20), IL1β, IL6, IL8, IL23A and tumor necrosis factor-α (TNF-α) cytokines in activated keratinocytes [30,42]. Similarly, Xie et al. reported that IR inhibited the expression of IL1, IL6, IL22, IL23 and IL17A in IMQ-induced psoriasis-like skin lesions [33]. Cheng et al. [30] showed that IN and tryptanthrin differentially reduced the expression of CCL20 chemokine, independent of NF-κB-p65-activation [30]. However, this contradicts the conclusions of Zhao et al. [47], who reported that IR strongly inhibits CCL20 expression and secretion in IL-17A-stimulated HaCaT cells by modulating the mitogen-activated protein kinase kinase kinase 7 (TAK1) signaling pathway, including NF-κB-p65 [47].

The inflammatory cytokine IL-17 plays a critical role in psoriasis pathogenesis. IL-17-secreting cells include T helper 17 cells (Th 17) and γδ T cells. Recent evidence has suggested that innate dermal γδ T cells are the primary cells that produce IL-17 [54–56]. IR can reduce the expression and secretion of IL-17 in IL-1β- and IL-23-induced-γδ T cells *via* the JAK3/STAT3 pathway [33].

In addition, infiltration of inflammatory cells also plays a crucial role in the pathogenesis of psoriasis. The migration of inflammatory cells from the circulatory system to skin is mediated by cell adhesion molecules. Vascular cell adhesion molecule-1 (VCAM-1) in the endothelium is important for the initial trafficking of memory T cells in psoriatic lesions [57]. IN can inhibit TNF-α-induced VCAM-1 expression, but not of the intercellular adhesion molecule-1 (ICAM-1), in human umbilical vein endothelial cells *via* inhibition of the AP-1/c-Jun pathway [38]. Another pathological mechanism in the development of psoriasis is the disturbance in epidermal barrier function [58]. IN ameliorates psoriatic plaques by increasing claudin-1 expression and re-establishes the function of tight junctions in human primary keratinocytes [43].

Keratinocyte hyperproliferation plays a pivotal role in the pathogenesis of psoriasis. IN and its main components, IR, indigo and tryptanthrin, exhibit anti-proliferative effects by causing G2/M or G0/G1 cell cycle arrest in epidermal keratinocytes or vascular endothelial cells, targeting proliferating cell nuclear antigen (PCNA), cyclin-dependent kinases (CDKs) and cyclins *via* multiple signaling pathways. IN and IR, in epidermal keratinocytes, can differentially suppress the expression of cell division cycle 25 B (CDC25B) at both, the mRNA and protein levels [40]. Tryptanthrin arrests the cell cycle at the G2/M phase and inhibits the expression of cyclin A, cyclin B and CDK 1 and 2, by blocking the Akt and focal adhesion kinase (FAK) pathways in human vascular endothelial cells [37]. Another critical cell cycle regulatory protein is PCNA, which traverses the cell cycle from the G1 to S phase. Lin et al. found that PCNA expression was substantially decreased in both IN-treated lesions and cultured keratinocytes *in vitro*. The major components, IR and indigo, can cause cell cycle arrest at the G0/G1 phase in a dose-dependent manner [44]. Similar results were observed in HaCaT cells treated with IR. IR exerts an anti-proliferative effect by promoting demethylation of Wnt inhibitory factor 1 (wif-1) and suppressing the Wnt/β-catenin signaling pathway, leading to cell cycle arrest at the G0/G1 phase [45].

Moreover, IR can also moderate keratinocyte differentiation. Liu et al. [45] demonstrated that IR could downregulate the mRNA expression levels of involucrin and the transcriptional activation of transglutaminase 1 (TGase 1) and keratin 17 to improve the differentiation of HaCaT cells [45].

**Figure 1.**
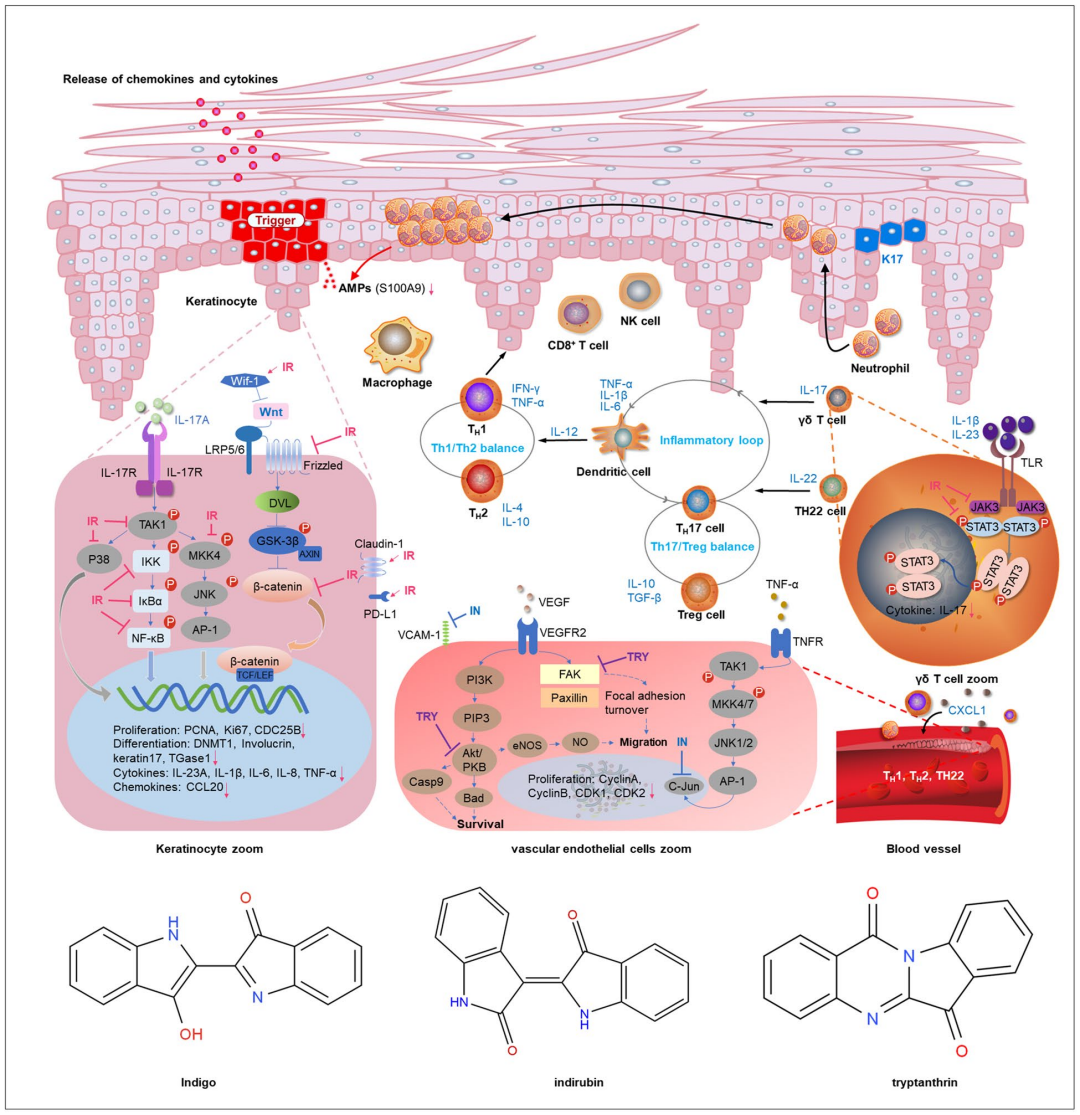
Diagram of the mechanism of indigo naturalis (IN) and its active components in psoriasis treatment using preclinical *in vitro* analysis. Diagram of the mechanisms of indigo naturalis (IN) and its active components in psoriasis treatment Indirubin (IR) inhibits the hyperproliferation and differentiation of keratinocytes (KCs) and expression of pro-inflammatory mediators. Moreover, IR suppressed the expression of IL-17 in γδ T-cells. IN and tryptanthrin have anti-proliferative and anti-angiogenic effects on vascular endothelial cells. The efficacy of indigo naturalis and its components may be attributed to the regulation of the mitogen-activated protein kinase kinase kinase 7 (TAK1), JAK3/STAT3, Wnt/β-catenin, Akt/PKB, focal adhesion kinase (FAK) and AP-1/c-Jun pathways.

Finally, anti-angiogenesis is an important strategy for the treatment of psoriasis. IN and tryptanthrin exert anti-angiogenic effects by inhibiting the migration and tube formation of vascular endothelial cells by blocking the Akt/PKB, FAK [37], or apelin pathways [39] (Figure 1).

## 5. Conclusion

Conventional treatment complemented with the use of IN enhances the therapeutic effects of psoriasis. As a single drug, indigo also has good therapeutic effect and few side effects. Multiple cell experiments have identified that IN, IR, indigo and tryptanthrin have anti-proliferative, anti-inflammatory and anti-angiogenic effects, *via* the regulation of the TAK1, JAK3/STAT3, Wnt/β-catenin, Akt/PKB, FAK and AP-1/c-Jun pathways.

## Supplementary Material

Supplemental Material

## Data Availability

The authors confirm that the data supporting the findings of this study are available within the article.

## References

[1] Armstrong AW, Mehta MD, Schupp CW, et al. Psoriasis prevalence in adults in the United States. JAMA Dermatol. 2021;157(8):940–946. doi: 10.1001/jamadermatol.2021.2007. 34190957 PMC8246333

[2] Vanderpuye-Orgle J, Zhao Y, Lu J, et al. Evaluating the economic burden of psoriasis in the United States. J Am Acad Dermatol. 2015;72(6):961.e5–967.e5. doi: 10.1016/j.jaad.2015.02.1099. 25882886

[3] Griffiths CEM, Armstrong AW, Gudjonsson JE, et al. Psoriasis. Lancet. 2021;397(10281):1301–1315. doi: 10.1016/S0140-6736(20)32549-6. 33812489

[4] Armstrong AW, Read C. Pathophysiology, clinical presentation, and treatment of psoriasis: a review. JAMA. 2020;323(19):1945–1960. doi: 10.1001/jama.2020.4006. 32427307

[5] Shear NH, Betts KA, Soliman AM, et al. Comparative safety and benefit-risk profile of biologics and oral treatment for moderate-to-severe plaque psoriasis: a network meta-analysis of clinical trial data. J Am Acad Dermatol. 2021;85(3):572–581. doi: 10.1016/j.jaad.2021.02.057. 33631216

[6] Lee C-L, Wang C-M, Kuo Y-H, et al. IL-17A inhibitions of indole alkaloids from traditional Chinese medicine Qing Dai. J Ethnopharmacol. 2020;255:112772. doi: 10.1016/j.jep.2020.112772. 32194230 PMC7156250

[7] Naganuma M, Sugimoto S, Mitsuyama K, et al. Efficacy of indigo naturalis in a multicenter randomized controlled trial of patients with ulcerative colitis. Gastroenterology. 2018;154(4):935–947. doi: 10.1053/j.gastro.2017.11.024. 29174928

[8] Yu H, Li T-N, Ran Q, et al. *Strobilanthes cusia* (nees) kuntze, a multifunctional traditional chinese medicinal plant, and its herbal medicines: a comprehensive review. J Ethnopharmacol. 2021;265:113325. doi: 10.1016/j.jep.2020.113325. 32889034

[9] Nguyen LTH, Oh TW, Choi MJ, et al. Anti-psoriatic effects and IL-22 targeting mechanism of indirubin by suppressing keratinocyte inflammation and proliferation. Appl Sci. 2021;11(24):11599. doi: 10.3390/app112411599.

[10] Chen H, Wang SP. Observation of the efficacy of compound Qingdai capsules in the treatment of psoriasis vulgaris and its effect on serum IL-2 and IL-8. J Chin Med Mater. 2004;27(11):885–886.

[11] Liang Y, Wang WC, Yi J, et al. Clinical observation of smilax glabra and Qingdai decoction for the treatment of psoriasis vulgaris (blood fever type) and detection of TNF-α and VEGF levels. Guangming J Chin Med. 2015;30:2132–2134.

[12] Wang WC, Liang Y, Yan ZR. Clinical reports of the treatment of 30 cases of psoriasis vulgaris with the decoction of smilax glabra and Qingdai. Liaoning J Tradit Chin Med. 2011;38(6):1135–1136.

[13] Cai WL, Yang XY, Yao SL, et al. Efficacy of combination of Qingdai capsules and acitretin on the treatment of psoriasis vulgaris and effect on serum levels of tumor necrosis factor-α, interleukin-17, and interleukin-23. China’s Naturopathy. 2021;29:89–91.

[14] Pang J, Ye TT, Shu ZX. Efficacy of compound Qingdai capsule in the treatment of psoriasis vulgaris and its effect on serum inflammatory factors. World Chin Med. 2017;12:1298–1301.

[15] Wu B, Su XJ, Jiang CH. Clinical observation of compound Qingdai capsules combined with thymus peptide enteric-coated tablets for the treatment of psoriasis vulgaris. Contemp Med. 2009;15:146.

[16] Xue H, Li JN, Zhang XL, et al. Clinical efficacy of compound qingdai capsules in the treatment of children’s drip psoriasis. Chin J Dermatovenereol Integr Tradit West Med. 2021;20:489–491.

[17] Zhang H, Zhang Y. The effect of compound Qingdai capsules in adjuvant treatment of psoriasis on clinical symptoms, immune function and clinical efficacy. Chin J Drug Abuse Prevent. 2021;27(5):763–766.

[18] Ji XH. Clinical observation on narrow -band ultraviolet B and compound Qingdai pill in treatment of psoriasis. Syst Med. 2017;2:87–89.

[19] Wang MZ. Efficacy of ultraviolet radiation combined with compound Qingdai pills in the treatment of psoriasis in 46 cases. Heilongjiang Med J. 2009;22(1):88–89.

[20] Xie CL, Fu MN, Shi N. Efficacy of laser therapy combined with compound Qingdai capsules in the treatment of psoriasis and its effect on serum TNF-α, IL-17, IL-23. Modern J Integr Tradit Chin West Med. 2017;26:2363–2365.

[21] Yang HR, Jiang YH, Deng Y. Qingdai compound capsule combined with ultraviolet irradiation on the elderly with psoriasis. Geriatrics Health Care. 2019;25:644–647.

[22] Han FH, Li SH, Li GD. Clinical study of compound Qingdai pill combined with compound Antaisu in treatment of psoriasis. Med Innov China. 2014;11:97–99.

[23] Han XS, Yin D, Tian K, et al. Efficacy of acitretin combined with compound Qingdai capsules in the treatment of psoriasis. Chin J Dermatovenereol Integr Tradit West Med. 2017;16:66–67.

[24] Lin LL, Suo WH. The effects of Qingdai Liangxue decoction combined with acitretin and Calcipotrio Ointmenon on psoriasis and its influence on serum cytokines (the effects of Qingdai Liangxue decoction combined with acitretin and Calcipotrio Ointmenon on psoriasis and its influence on serum cytokines). J Hubei Univ Chin Med. 2017;19(5):75–78.

[25] Lin YL. Effect of compound Qingdai pills in treating moderate and severe psoriasis vulgaris. J Dermatol Venereol. 2018;40:836–837.

[26] Yang C, Lin JX, Wang W, et al. The effect of compound Qingdai capsules combined with calcipotriene betamethasone cream in treating psoriasis about serum IL-17、TNF-α、IL-23. Chin J Dermatovenereol Integr Tradit West Med. 2019;18:602–604.

[27] Chen LH. Observation of the efficacy of compound Qingdai cream in the treatment of psoriasis vulgaris. J Gansu Univ Chin Med. 2010;27:45–47.

[28] Lin YK, Wu YH, Yang RJ, et al. Evaluation of the clinical efficacy of compound Qingdai oil in the treatment of psoriasis. J Chengdu Univ TCM. 2006;29(2):13–16.

[29] Chen LH, Zhao WJ. Compound Qingdai cream treated 60 cases of psoriasis vulgaris. Guangming J Chin Med. 2014;29(7):1413–1414.

[30] Cheng HM, Kuo YZ, Chang CY, et al. The anti-TH17 polarization effect of indigo naturalis and tryptanthrin by differentially inhibiting cytokine expression. J Ethnopharmacol. 2020;255:112760. doi: 10.1016/j.jep.2020.112760. 32173427

[31] He E, Li H, Li X, et al. Transdermal delivery of indirubin-loaded microemulsion gel: preparation, characterization and anti-psoriatic activity. Int J Mol Sci. 2022;23(7):3798. doi: 10.3390/ijms23073798. 35409158 PMC8998921

[32] Wang Q. Study on the effect of indigo naturalis oil on the model of psoriasis lesions [master’s thesis]. Xiamen(FJ): Fujian Medical University; 2018.

[33] Xie XJ, Di TT, Wang Y, et al. Indirubin ameliorates imiquimod-induced psoriasis-like skin lesions in mice by inhibiting inflammatory responses mediated by IL-17A-producing γδ T cells. Mol Immunol. 2018;101:386–395. doi: 10.1016/j.molimm.2018.07.011. 30064075

[34] Xie XJ. Effects of Liangxue-Jiedu prescription and Indigo Naturalis on KC/γδT activation in the in vitro and in vitro psoriasis model including CCL20 [master’s thesis]. Beijing: Beijing University of Chinese Medicine; 2017.

[35] Xue X, Wu J, Li J, Xu J, Dai H, Tao C, Li C, Hu J. Indirubin attenuates mouse psoriasis-like skin lesion in a CD274-dependent manner: an achievement of RNA sequencing. Biosci Rep. 2018;38(6):BSR20180958. doi: 10.1042/BSR20180958. PMC6250808 30341238

[36] Xue XC. Function and regulation of PD-L1 in keratinocytes and intervention of indirubin [dissertation]. Shanghai: The Second Military Medical University; 2019.

[37] Chang HN, Huang ST, Yeh YC, et al. Indigo naturalis and its component tryptanthrin exert anti-angiogenic effect by arresting cell cycle and inhibiting Akt and FAK signaling in human vascular endothelial cells. J Ethnopharmacol. 2015;174:474–481. doi: 10.1016/j.jep.2015.08.050. 26341616

[38] Chang HN, Pang JHS, Yang SH, et al. Inhibitory effect of indigo naturalis on tumor necrosis factor-α-induced vascular cell adhesion molecule-1 expression in human umbilical vein endothelial cells. Molecules. 2010;15(9):6423–6435. doi: 10.3390/molecules15096423. 20877233 PMC6257747

[39] Chang HN, Yeh YC, Chueh HY, et al. The anti-angiogenic effect of tryptanthrin is mediated by the inhibition of apelin promoter activity and shortened mRNA half-life in human vascular endothelial cells. Phytomedicine. 2019;58:152879. doi: 10.1016/j.phymed.2019.152879. 31005035

[40] Hsieh WL, Lin YK, Tsai CN, et al. Indirubin, an acting component of indigo naturalis, inhibits EGFR activation and EGF-induced CDC25B gene expression in epidermal keratinocytes. J Dermatol Sci. 2012;67(2):140–146. doi: 10.1016/j.jdermsci.2012.05.008. 22721997

[41] Lee CL, Wang CM, Hu HC, et al. Indole alkaloids indigodoles A-C from aerial parts of *Strobilanthes cusia* in the traditional Chinese medicine Qing Dai have anti-IL-17 properties. Phytochemistry. 2019;162:39–46. doi: 10.1016/j.phytochem.2019.02.016. 30852259

[42] Li QW, Liu SG, Lin JX, et al. Effect of indirubin on the expression of proinflammatory cytokines in keratinocytes. J Diagn Ther Dermato-Venereol. 2021;28:182–185.

[43] Lin YK, Chen HW, Leu YL, et al. Indigo naturalis up-regulates claudin-1 expression in cultured human keratinocytes and psoriatic lesions. J Ethnopharmacol. 2013;145(2):614–620. doi: 10.1016/j.jep.2012.11.044. 23220199

[44] Lin YK, Leu YL, Yang SH, et al. Anti-psoriatic effects of indigo naturalis on the proliferation and differentiation of keratinocytes with indirubin as the active component. J Dermatol Sci. 2009;54(3):168–174. doi: 10.1016/j.jdermsci.2009.02.007. 19303259

[45] Liu SG, Luo GP, Qu YB, et al. Indirubin inhibits wnt/β-catenin signal pathway via promoter demethylation of WIF-1. BMC Complement Med Ther. 2020;20(1):250. doi: 10.1186/s12906-020-03045-9. 32795328 PMC7427955

[46] Wang H, Sun LY, Deng BX, et al. The effects of shikonin and indirubin on apoptosis of cultured keratinocytes. Clin J Lepr Skin Dis (Chin). 2003;19(4):336–338.

[47] Zhao J, Xie X, Di T, et al. Indirubin attenuates IL-17A-induced CCL20 expression and production in keratinocytes through repressing TAK1 signaling pathway. Int Immunopharmacol. 2021;94:107229. doi: 10.1016/j.intimp.2020.107229. 33611057

[48] Lin YK, See LC, Huang YH, et al. Efficacy and safety of indigo naturalis extract in oil (lindioil) in treating nail psoriasis: a randomized, observer-blind, vehicle-controlled trial. Phytomedicine. 2014;21(7):1015–1020. doi: 10.1016/j.phymed.2014.02.013. 24680615

[49] Cheng HM, Wu YC, Wang Q, et al. Clinical efficacy and IL-17 targeting mechanism of indigo naturalis as a topical agent in moderate psoriasis. BMC Complement Altern Med. 2017;17(1):439. doi: 10.1186/s12906-017-1947-1. 28865459 PMC5581407

[50] Lin YK, Chang CJ, Chang YC, et al. Clinical assessment of patients with recalcitrant psoriasis in a randomized, observer-blind, vehicle-controlled trial using indigo naturalis. Arch Dermatol. 2008;144(11):1457–1464. doi: 10.1001/archderm.144.11.1457. 19015420

[51] Lin YK, Chang YC, Hui RC, et al. A Chinese herb, indigo naturalis, extracted in oil (lindioil) used topically to treat psoriatic nails: a randomized clinical trial. JAMA Dermatol. 2015;151(6):672–674. doi: 10.1001/jamadermatol.2014.5460. 25738921

[52] Wang ZC. A randomized, double-blind study of compound Qingdai capsules for the treatment of psoriasis (a randomized double-blinded study on treatment of psoriasis with composit indigofera suffroticosa). China’s Naturopathy. 2003;11:48–49.

[53] Yazdanpanah MJ, Vahabi-Amlashi S, Pishgouy M, et al. Comparing the topical preparations of indigo naturalis from Chinese and Iranian origin in the treatment of plaque-type psoriasis: a preliminary randomized double-blind pilot study. Eur J Integr Med. 2021;43:101310. doi: 10.1016/j.eujim.2021.101310.

[54] Akitsu A, Iwakura Y. Interleukin-17-producing γδ T (γδ17) cells in inflammatory diseases. Immunology. 2018;155(4):418–426. doi: 10.1111/imm.12993. 30133701 PMC6231014

[55] Cai Y, Shen X, Ding C, et al. Pivotal role of dermal IL-17-producing γδ T cells in skin inflammation. Immunity. 2011;35(4):596–610. doi: 10.1016/j.immuni.2011.08.001. 21982596 PMC3205267

[56] Sutton CE, Mielke LA, Mills KH. IL-17-producing γδ T cells and innate lymphoid cells. Eur J Immunol. 2012;42(9):2221–2231. doi: 10.1002/eji.201242569. 22949320

[57] Wakita H, Takigawa M. E-selectin and vascular cell adhesion molecule-1 are critical for initial trafficking of helper-inducer/memory T cells in psoriatic plaques. Arch Dermatol. 1994;130(4):457–463. doi: 10.1001/archderm.1994.01690040061008. 7513146

[58] Chung E, Cook PW, Parkos CA, et al. Amphiregulin causes functional downregulation of adherens junctions in psoriasis. J Invest Dermatol. 2005;124(6):1134–1140. doi: 10.1111/j.0022-202X.2005.23762.x. 15955087

