# Supplementary material for: Evidence and potential mechanism of action of indigo naturalis and its active components in the treatment of psoriasis
Source: Ann Med. 2024 Sep 24;56(1):2329261. doi: 10.1080/07853890.2024.2329261 (PMC11423532; doi:10.1080/07853890.2024.2329261)
Supplement: Supplemental Material [file IANN_A_2329261_SM4652.zip › Supplementary Table S1.docx]

| **Table S1. The characteristics of included clinical studies** | | | | | | | | | | | | |
| --- | --- | --- | --- | --- | --- | --- | --- | --- | --- | --- | --- | --- |
| **Author Year** | **Sample Size** | | **Average Age (years)** | | **Gender (M/F)** | | **Duration of Psoriasis (year)** | | **Intervention** | | **Course of treatment (week）** | **Outcome** |
|  |  |  | **(Mean ± SD)** | |  |  | **(Mean ± SD)** | |  |  |  |  |
|  | **E** | **C** | **E** | **C** |  | | **E** | **C** | **E** | **C** |  |  |
| Zhang, et al., 2021 | 60 | 60 | 57.04 ± 18.13 | 56.36 ± 17.29 | | 63/57 | 8.45 ± 2.32 | 8.38 ± 2.71 | IND + OOD | OOD | 8W | PASI |
| Yang, et al., 2019 | 58 | 58 | 68.7 ± 8.8 | 69.1 ± 9.2 | | 77/39 | 6.2 ± 1.3 | 6.1 ± 1.1 | IND + LT | LT | 8W | PASI, AEs |
| Pang, et al., 2017 | 45 | 45 | 36. 48 ± 14. 21 | 37. 02 ± 44. 47 | | 46/44 | 47. 68 ± 18. 22M | 49. 13 ± 18. 80M | IND + OOD | OOD | 8W | PASI |
| Ji, 2017 | 52 | 52 | 40.8 ± 1.3 | 39.4 ± 1.6 | | 60/44 | 5.4 ± 1.1 | 5.1 ± 0.7 | IND + LT | LT | 8W | PASI, AEs |
| Lin, et al., 2017 | 31 | 31 | 35.4 ± 6.6 | 35.6 ± 6.4 | | 23/39 | 1.12 ± 0.28 | 1.14 ± 0.26 | IND + OOD + OTT | OOD + OTT | 4W | PASI |
| Han, et al., 2017 | 48 | 48 | 45.72 ± 5.78 | 43.56 ± 4.43 | | 53/43 | 3.5 | 4.2 | IND + OOD + OTT | OOD + OTT | 8W | PASI, AEs |
| Liang, et al., 2015 | 30 | 30 | 34.28 ± 10.26 | 33.46 ± 10.12 | | 34/26 | 6.8 | 5.6 | IND | OOD | 4W | PASI |
| Chen, et al., 2014 | 20 | 20 | 40.5 | 41.6 | | 23/17 | 8.5 | 9.3 | INT | OTT | 4W | PASI |
| Wang, et al., 2010 | 30 | 30 | 35.24 ± 10.28 | 33.48 ± 10.02 | | 33/27 | 6.5 | 5.4 | IND | OOD | 8W | PASI |
| Chen, 2010 | 60 | 56 | 39.5 | 41.6 | | 76/39 | 8.7 | 9.3 | INT + OOD | OOD | 8W | PASI |
| Wang, 2003 | 25 | 27 | 26.68 ± 12.3 | 8.62 ± 14.23 | | 36/16 | 56.68 ± 40.12M | 61.24 ± 39.62M | IND | PBO | 4W | PASI, AEs |
| Lin, et al., 2005 | 48 | 48 | 34.74 ± 11.08 | 34.74 ± 11.08 | | 41/16 | 10.81 ± 7.42 | 10.81 ± 7.42 | INT | PBO | 12W | PSI, AEs |
| Wang, 2008 | 44 | 43 | 39.74 | 39.74 | | 50/37 | 9.34 | 8.67 | IND + LT | LT | 6W | PASI, AEs |
| Han, et al., 2014 | 51 | 50 | 33.1 ± 4.6 | 34.5 ± 4.2 | | 56/45 | 3.6 ± 1.3 | 3.4 ± 1.6 | IND + OOD + OTT | OOD + OTT | 8W | PASI, AEs |
| Xue, et al., 2021 | 34 | 34 | 7.8 ± 2.3 | 7.7 ± 2.1 | | 29/39 | 13.4 ± 5.3D | 13.9 ± 4.8D | IND + OOD | OOD | 8W | PASI, AEs |
| Cai, et al., 2021 | 33 | 33 | 36.9 ± 4.7 | 36.9 ± 4.7 | | 36/30 | 13.7 ± 4.1 | 13.7 ± 4.1 | IND + OOD | OOD | 6W | PASI, AEs |
| Yang, et al., 2019 | 41 | 41 | 38.24 ± 4.19 | 38.57 ± 4.03 | | 49/33 | 14.39 ± 2.78 | 14.79 ± 1.93 | IND + OTT | OTT | 8W | PASI, AEs |
| Lin, 2018 | 50 | 50 | 34.9 ± 5.3 | 36.8 ± 4.6 | | 49/51 | 5.9 ± 2.1 | 6.1 ± 1.7 | IND + OOD + OTT | OOD + OTT | 8W | PASI, AEs |
| Xie, et al., 2017 | 49 | 48 | 37.6 ± 5.4 | 37.6 ± 5.4 | | 66/31 | 15.4 ± 3.8 | 15.4 ± 3.8 | IND + LT | LT | 6W | PASI, AEs |
| Wu, et al., 2009 | 60 | 50 | 36.5 | 34.6 | | 38/22 | 18M | 20M | IND + OOD | OOD | 8W | PASI, AEs |
| Chen, et al., 2004 | 61 | 55 | 37.51 ± 11.32 | 35.13 ± 10.91 | | 75/41 | 5.16 ± 5.02 | 4.87 ± 4.71 | IND | OOD | 8W | PASI, AEs |
| Lin, et al., 2014 | 30 | 30 | 40.7 ± 12.6 | 40.7 ± 12.6 | | 24/7 | 5.2 ± 5.5 | 5.2 ± 5.5 | INT | PBO | 12W | shNAPSI |
| Yazdanpanah, et al., 2021 -1 | 10 | 10 | 33.08 ± 10.92 | 31.6 ± 8.98 | | N/A | N/A | N/A | INT | OTT | 8W | PASI, AEs |
| Yazdanpanah, et al., 2021 -2 | 10 | 10 | 41.5 ± 10.98 | 31.6 ± 8.98 | | N/A | N/A | N/A | INT | OTT | 8W | PASI, AEs |
| Cheng, et al., 2017 | 16 | 8 | 39.3 ± 10.1 | 40.1 ± 10.9 | | 17/7 | 13.1 ± 11.1 | 14.9 ± 12.1 | INT | PBO | 8W | PASI, AEs |
| Lin, et al., 2018 | 34 | 34 | 34.6 ± 11.5 | 34.6 ± 11.5 | | 32/10 | 10 | 10 | INT | PBO | 12W | PSI, AEs |
| Lin, et al., 2015 | 33 | 33 | 41.9 ± 9.4 | 41.9 ± 9.4 | | 22/11 | 5.8 ± 6.4 | 5.8 ± 6.4 | INT | OTT | 24W | shNAPSI |

**Abbreviation:** E, experimental groups, C, control groups; IND, decoctions containing Indigo Naturalis (IN) and TCM formulas with IN as the sovereign herb; OOD, other oral drugs; LT, light therapy; OTT, other topical therapies; PBO, placebo; PASI, Psoriasis Area and Severity Index; shNAPSI, single hand Nail Psoriasis Severity Index; PSI, Psoriasis Severity Index; AEs, adverse events; W, week; M, month; D, day; N/A, not applicable.
