# Supplementary material for: Evidence and potential mechanism of action of indigo naturalis and its active components in the treatment of psoriasis
Source: Ann Med. 2024 Sep 24;56(1):2329261. doi: 10.1080/07853890.2024.2329261 (PMC11423532; doi:10.1080/07853890.2024.2329261)
Supplement: Supplemental Material [file IANN_A_2329261_SM4652.zip › Supplementary Table S2.docx]

| **Table S2. Checklist of Indigo Naturalis and its main components in preclinical researches** | | | | | | | | |
| --- | --- | --- | --- | --- | --- | --- | --- | --- |
| **Category** | | **Study** | **Researched Medicine** | **Access** | **Route of administration** | **Experimental concentration** | **Dose** | **Preparation method** |
| Preclinical research | *in vivo* | Xue et al., 2018 | IR | N/A | s.c. | N/A | 50 mg/kg/d | N/A |
|  |  | Xie et al., 2018 | IR | National Institutes for Food and Drug Control, China | i.g. | N/A | 50, 25, 12.5mg/kg/d | dissolved in dimethyl sulfoxide (DMSO), then diluted with normal saline to achieve a final DMSO concentration of < 0.1% |
|  |  | He et al., 2022 | Indirubin-Loaded Microemulsion Gel | N/A | ext. | N/A | N/A | N/A |
|  |  | Xue, 2019 | IR | China Institute of Food and Drug Control | s.c. | N/A | 50 mg/kg/d | dissolved in dimethyl sulfoxide (DMSO) |
|  |  | Xie, 2017 | IR | China Institute of Food and Drug Control | i.g. | N/A | 50, 25, 12.5mg/kg/d | N/A |
|  |  | Wang, 2018 | IN oil | Beijing Tongrentang | ext. | 1∶10（W/V） | 0.1 mL/cm2 | dissolved in olive oil or camellia oil |
|  |  | Cheng, 2020 | IN ointment | N/A | ext. | 10 mg/cm2 | N/A | N/A |
|  |  | Nguyen et al.,2021 | IR | Sigma-Aldrich (St. Louis, MO, USA) | ext. | 0.1% and 1% | 20 µL | dissolved in TFA/acetonitrile |
|  | *in vitro* | Cheng, 2020 | IN, indigo, indirubin and tryptanthrin | Kaiser Pharmaceutical Co.Ltd (Batch number G21617) Sigma Aldrich (St. Louis, Missouri, USA) | N/A | Indigo naturalis (0–200 μg/mL) tryptanthrin (0-25μM) | N/A | dissolved in dimethyl sulfoxide (DMSO) |
|  |  | Lee et al., 2021 | IR | Beijing Solebao Technology Co., Ltd | N/A | 0.5 μM,1.0 μM,2.0 μM | N/A | N/A |
|  |  | Xue, 2019 | IR | China Institute of Food and Drug Control | N/A | 5μg/mL | N/A | dissolved in dimethyl sulfoxide (DMSO) |
|  |  | Xie, 2017 | IR | China Institute of Food and Drug Control | N/A | 16μM, 8μM, 2μM | N/A | dissolved in phosphate buffer solution (PBS) |
|  |  | Liu et al., 2020 | IR | Aladdin (Shanghai, China) | N/A | different concentrations (0, 0.04 μM, 0.2 μM, and 1 μM) | N/A | dissolved in dimethyl sulfoxide (DMSO) |
|  |  | Lee, 2020 | Indigodole D, cephalandole B | Sheng Chang Pharmaceutical Co., Ltd. in Zhongli District, Taoyuan City, Taiwan | N/A | Indigodole D (0-50 μg/mL) cephalandole B (0-50 μg/mL) | N/A | N/A |
|  |  | Zhao et al., 2021 | IR | National Institutes for Food and Drug Control (Beijing, China) | N/A | N/A | 0.5 to 64 μM | dissolved in dimethyl sulfoxide (DMSO), and then diluted with saline to achieve a final DMSO concentration of < 0.1% |
|  |  | Chang et al., 2019 | tryptanthrin | Sigma | N/A | N/A | 0-50μM | dissolved in 100% dimethyl sulfoxide (DMSO) to give 10mM stock solution. |
|  |  | Lin et al., 2013 | IN, IR | IN: Guang Sheng Trading (Taipei, Taiwan) IR: indirubin was obtained from Alexis (Lausen, Switzerland) | N/A | IN（0-500μg/mL） IR（0-20μg/mL） | N/A | dissolved in DMSO |
|  |  | Lee, 2019 | Indole alkaloids indigodoles A–C | N/A | N/A | N/A | indigodoles A (0-22μM) indigodoles B (0-36μM) indigodoles C (0-360μM) | N/A |
|  |  | Wang et al., 2003 | IR | China Institute of Pharmaceutical and Biological Products Inspection | N/A | 0.1, 0.25, 0.5 mmol/ml | 26, 65, 130 μg | dissolved in dimethyl sulfoxide (DMSO) |
|  |  | Chang, 2015 | IN, tryptanthrin | IN: Guang Sheng Trading (Taipei, Taiwan) tryptanthrin: Sigma | N/A | IN: 0, 10, 50, 250, 500, 1000μg/ ml tryptanthrin: 0, 10, 25, 50μM | N/A | dissolved in dimethyl sulfoxide (DMSO) |
|  |  | Hsieh, 2012 | IN, IR | IN: Guang Sheng Trading (Taipei, Taiwan) IR: Alexis | N/A | IN: 0, 125, 250, 500μg/ml IR: 0, 1, 5, 10, 20μM | N/A | dissolved in dimethyl sulfoxide (DMSO) |
|  |  | Chang, 2010 | IN | Guang Sheng Trading (Taipei, Taiwan) | N/A | 500μg/ mL | N/A | dissolved in dimethyl sulfoxide (DMSO) |
|  |  | Lin, 2009 | IN | IN: Guang Sheng Trading (Taipei, Taiwan) Indigo:Fluka IR:Alexis | N/A | IN: 0, 10, 50, 250, 500μg/ ml Indigo:0, 5, 25, 50, 100μM IR:0, 5, 25, 50, 100μM | N/A | dissolved in dimethyl sulfoxide (DMSO) |
|  |  | Ly et al.,2021 | IR, indigo, and tryptanthrin | Sigma-Aldrich (St. Louis, MO, USA) | N/A | IR: 30µM  Indigo: 30µM  tryptanthrin: 30µM | N/A | dissolved in TFA/acetonitrile |

**Abbreviations:** IN, indigo naturalis; IR, indirubin; N/A, not applicable; i.g., intragastric administration; ext., external; s.c., subcutaneous injection.
