# Supplementary material for: Evidence and potential mechanism of action of indigo naturalis and its active components in the treatment of psoriasis
Source: Ann Med. 2024 Sep 24;56(1):2329261. doi: 10.1080/07853890.2024.2329261 (PMC11423532; doi:10.1080/07853890.2024.2329261)
Supplement: Supplemental Material [file IANN_A_2329261_SM4652.zip › Supplementary Table S3.docx]

| **Table S3. The characteristics of preclinical studies in vitro** | | | | |  |
| --- | --- | --- | --- | --- | --- |
| **Author, year** | **Cell Type** | **Cell origin (species)** | **Cell Tracking Method** | **Outcomes** | **Pathway** |
| Cheng et al, 2020 | aHK | Adult keratinocytes from skin | MTS kits (The number of viable cells)  IC50  PCR (S100A9, CCL20, IL23A, IL1B, IL8, IL6, TNFA)  WB (FLAG/MYC-tagged and endogenous RORγT, RORC-expressing Jurkat T cells)  RT-qPCR (IL17A, IL17F, IL22, CCR6, IL23R and CCL20 in RORC-bearing Jurkat T cells) | IN differentially inhibited the proliferation of keratinocytes and endothelial cells but not monocytes, fibroblasts nor Jurkat T cells. Tryptanthrin was the most potent compound to reduce their proliferation. | N/A |
|  | HMEC-1 | N/A |  |  |  |
|  | Human Jurkat T | N/A |  |  |  |
|  | Monocytic U937 lines | N/A |  |  |  |
|  | Human skin WS1 fibroblasts | N/A |  |  |  |
| Li et al,  2021 | HaCaT | Human normal skin immortalized KC | ELISA (IL-1β, IL-6, TNF-α)  PT-PCR (IL-1β, IL-6, TNF-α mRNA) | Indirubin can lower expression levels of pro-inflammatory cytokines such as IL-1β, IL-6, and TNF-α in keratinocytes, leading to the improvements of psoriasis． | N/A |
| Xue et al, 2019 | HaCaT | Human primary epidermal KC | PCR (PD-L1 mRNA) | Indirubin is a multi-targeted drug that can alleviate inflammatory responses by regulating PD-L1 expression in epidermal keratinocytes. And the regulation was carried by affecting the function of  miR-15a-5P. | miR-15a-5P pathway |
| Xie et al, 2017 | HaCaT | Human normal skin immortalized KC | CCK8(cell viability-OD)  ELISA (CCL20) PCR (CCL20 mRNA) | Indirubin can lead to the down-regulation of CCL20 expression and secretion in HaCaT cells, which is one of the mechanisms for the treatment of psoriasis. | N/A |
|  | γδT cell | Mouse spleen cells | CCK8 (cell viability-OD)  ELISA (IL-17A)  PCR (IL-17 mRNA)  WB (P-STAT3, P-JAK3) | Indirubin may inhibit IL-17+γδT cell mediated inflammatory responses via JAK/STAT3 mediated signaling pathway. | JAK/STAT3 pathway |
| Wang et al, 2003 | Keratinocytes Colo-16 | N/A | FCM (the content of hypodiploid in the cells)  AnnexinV (phosphatidylserine) | Indirubin in some concentration could induce apoptosis. | N/A |
| Liu et al, 2020 | HaCaT | Human normal skin immortalized KC | WB (DNMT1, wif-1, Frizzled2, Frizzled5, and β-catenin)  PCR (DNMT1, wif-1, Frizzled2, Frizzled5, and β-catenin)  ELISA (DNMT1, wif-1)  MSP (wif-1 promoter methylation status)  CCK8 (cell viability-OD)  WB (Involucrin, Loricrin, Filaggrin, Keratin 17)  PCR (Involucrin, Loricrin, Filaggrin, Keratin 17, TGase1)  Annexin V-FITC and PI (apoptotic cells)  FCM (cell cycle) | Indirubin promoted the demethylation of wif-1 and suppressed the wnt/β-catenin signal pathway, thereby exerted an anti-proliferative effect. | Wnt/β-catenin signal pathway |
| Lee et al, 2020 | CD4+ lymphocytes  polarized Th17 cells | Primary mouse CD4+ lymphocytes | FCM (IL-17A secretion cells)  PI (cell viability)  Microplate Reader (luminescence) | Indole alkaloids, indigodoles A, C, D, tryptanthrin, and indirubin could contribute to anti-IL 17A properties of Qing Dai. | N/A |
| Zhao et al, 2021 | HaCaT | Human normal skin immortalized KC | CCK-8 (cell viability)  RNA-seq analysis  PCR (Ccl20 mRNA, IL-17RA)  ELISA (Ccl20 protein)  WB (p65, IKK, IκBα, p38, MKK4 and TAK1) | Indirubin treatment strongly inhibited CCL20 expression and secretion in IL-17A stimulated HaCaT cells. | TAK1 signaling pathway |
| Chang et al, 2019 | HUVECs | Human umbilical vein endothelial cells | PCR (apelin, PCSK3, APJ, apelin mRNA half-life (t1/2))  ELISA (apelin, apelin-13)  WB (PCSK3, APJ)  Transwell filter (the mean number of migrating cells)  ECM gel-induced capillary tube formation (The percentages of capillary-like tubes of cells)  dual luciferase assay (the promoter activity of apelin)  the percentage of apelin mRNA of HUVECs | The anti-angiogenic effect of tryptanthrin was mediated by down-regulating apelin gene expression through suppression of promoter activity and decrease of mRNA stability in human vascular endothelial cells. | apelin pathway |
| Lee et al, 2019 | CD4+ lymphocytes  Jurkat cells | Naïve mouse CD4+ lymphocytes | FCM (IL-17+ cells)  Microplate Reader (IL-17Luc cells luminescence) | Indigodole C and tryptanthrin could significantly inhibit IL-17 production of Th17 cells. In addition, indigodole A and indirubin showed notably anti-IL-17 gene expression in dose-dependent effects without cytotoxicity toward Th17 and Jurkat cells, respectively. | IL-17 targeting signal |
| Chang et al, 2015 | HVEC | Human umbilical cord veins | CAM (angiogenesis index)  MTT (Vascular endothelial cell count)  RT-PCR (Cyclin D, Cyclin E, Cyclin A, Cyclin B, CDK1, CDK2)  WB (Akt, FAK, p-Akt, p-FAK)  Tube Formation Assay and Cell Migration  FCM (cell cycle) | Indigo naturalis and tryptanthrin inhibited the in vivo vascular endothelial growth.  Tryptanthrin resulted in a cell cycle arrest and could reduce the phosphorylated levels of both protein kinase B (PKB or Akt) and focal adhesion kinase (FAK). | Akt and FAK pathway |
| Lin et al, 2013 | Human keratinocytes | Human newborn foreskin | MTT (TEER)  Immunofluorescence method (claudin-1)  RT-PCR (claudin-1 mRNA)  WB (claudin-1, Tubulin, PKC, p-PKC substrate, α-Tubulin) | Indigo naturalis up-regulates claudin-1 expression and restores TJ function in keratinocytes.  Indigo naturalis increased the activity of protein kinase C (PKC). | PKC pathway |
| Hsieh et al, 2012 | aHEKs | Human skin | MTT (cell growth rate)  RT-PCR (CDC25B mRNA)  WB (CDC25B, Tubulin, p-EGFR, EGFR)  RT-PCR (CDC25B mRNA) | Indigo naturalis and indirubin down-regulate CDC25B expression at both the mRNA and protein levels. | N/A |
| Chang et al, 2010 | HUVECs | Human umbilical vein | LDH release assay (Cytotoxicity assay)  Adhesion of Jurkat T cell to HUVEC  Immunofluorescence method (ICAM-1, VCAM-1)  RT-PCR (ICAM mRNA, VCAM mRNA)  WB (VCAM-1, ICAM-1) | Indigo naturalis reduced TNF-α-induced increase in Jurkat T cell adhesion to HUVECs as well as decreased the protein and mRNA expression levels of VCAM-1 on HUVECs. | AP-1/c-Jun  pathway |
| Lin et al, 2009 | Human keratinocytes | Human skin | MTT (number of keratinocytes)  FCM (G0/G1, S, G2/M)  Immunofluorescence method (PCNA)  RT-PCR (involucrin gene)  WB (involucrin) | Indigo naturalis can decreased PCNA and increased involucrin | N/A |
| Nguyen et al.,2021 | HaCaT | N/A | XTT (viability of HaCaT cells)  ELISA (ICAM-1, TNF-α, IL-6, and IL-8)  Oxidative stress kit (ROS-positive cells)  DCF-DA assay (ROS production)  Ki67 proliferation kit (Ki67)  BrdU assay kit (ERK, STAT3)  Cell cycle kit (the proportion of cells entering each cell cycle)  WB (K16, Ki67, Cyclin D1,Cyclin E1, p-STAT3, STAT3, p-ERK, ERK, p-p38, p38, p-JNK, JNK)  Immunofluorescence method (p-ERK, p-STAT3) | Indirubin may have therapeutic potential in an IL-22-induced psoriasis model. | IL-22 pathway |

**Abbreviations**: IN, indigo naturalis; IR, indirubin; KC, keratinocyte cell; aHK, adult human keratinocytes from skin; HMEC-1, human immortalized microvascular endothelial cell line; IC50, the inhibitory concentration at 50% killing; IL, interleukin; TNFA, tumor necrosis factor A; WB, Western Bolt; RT-qPCR: quantitative real-time polymerase chain reaction; HaCaT, human immortalized keratinocyte; ELISA, enzyme-linked immunosorbent assay; PD-L1, programmed cell death ligand 1; CCK8, Cell Counting Kit-8; FCM, Flow Cytometry; CCL, C-C motif ligand; S100A, S100 calcium-binding protein A; DNMT1, DNA methyltransferase 1; wif-1, wnt inhibitory factor 1;PI, propidium iodide; IKK, inhibitor of nuclear factor kappa-B kinase; IκBα, NF-kappa-B inhibitor alpha; p65, transcription factor p65; p38, p38 MAP kinase; MKK4, mitogen-activated protein kinase kinase 4; TAK1, mitogen-activated protein kinase kinase 7; HUVECS, Human Umbilical Vein Endothelial Cells; PCSK3, proprotein convertase subtilisin/kexin 3; APJ, apelin receptor; HVEC, human vascular endothelial cells; CAM, embryonic chick chorioallantoic membrane assay; Akt or PKB, protein kinase B; FAK, focal adhesion kinase; TEER, transepithelial electrical resistance; PKC, protein kinase C; adult (HEKs), Human Epidermal Keratinocytes; EGFR, epithelial growth factor receptor; VCAM-1, vascular cell adhesion molecule-1; ICAM-1, intercellular adhesion molecule-1; PCNA, Proliferating Cell Nuclear Antigen; MSP, DNA methylation-specific PCR; VEGF, vascular endothelial growth factor; TGase1, transcriptional activation of transglutaminase 1.
