# Supplementary material for: Evidence and potential mechanism of action of indigo naturalis and its active components in the treatment of psoriasis
Source: Ann Med. 2024 Sep 24;56(1):2329261. doi: 10.1080/07853890.2024.2329261 (PMC11423532; doi:10.1080/07853890.2024.2329261)
Supplement: Supplemental Material [file IANN_A_2329261_SM4652.zip › Supplementary Table S4.docx]

| **Supplementary Table S4. Analysis of ear thickness in preclinical studies in vivo.** | | | | |
| --- | --- | --- | --- | --- |
| **Study** | **Change from Baseline** | | **Mean Difference** | ***p* value** |
|  | **(Mean ± SD)** | | **[95%CI]** |  |
|  | **Experiment** | **Control** |  |  |
| Nguyen et al.,2021-1 | 0.27 ± 0.01 | 0.31 ± 0.03 | -0.04 [-0.07; -0.01] | - |
| Nguyen et al.,2021-2 | 0.25 ± 0.01 | 0.31 ± 0.03 | -0.06 [-0.09; -0.03] | - |
| Total (95% CI) Random-effects I^2^ = 0% | | | -0.05 [-0.07; -0.03] | < 0.0001 |
