# Supplementary figures and images for: Evidence and potential mechanism of action of indigo naturalis and its active components in the treatment of psoriasis
Source: Ann Med. 2024 Sep 24;56(1):2329261. doi: 10.1080/07853890.2024.2329261 (PMC11423532; doi:10.1080/07853890.2024.2329261)

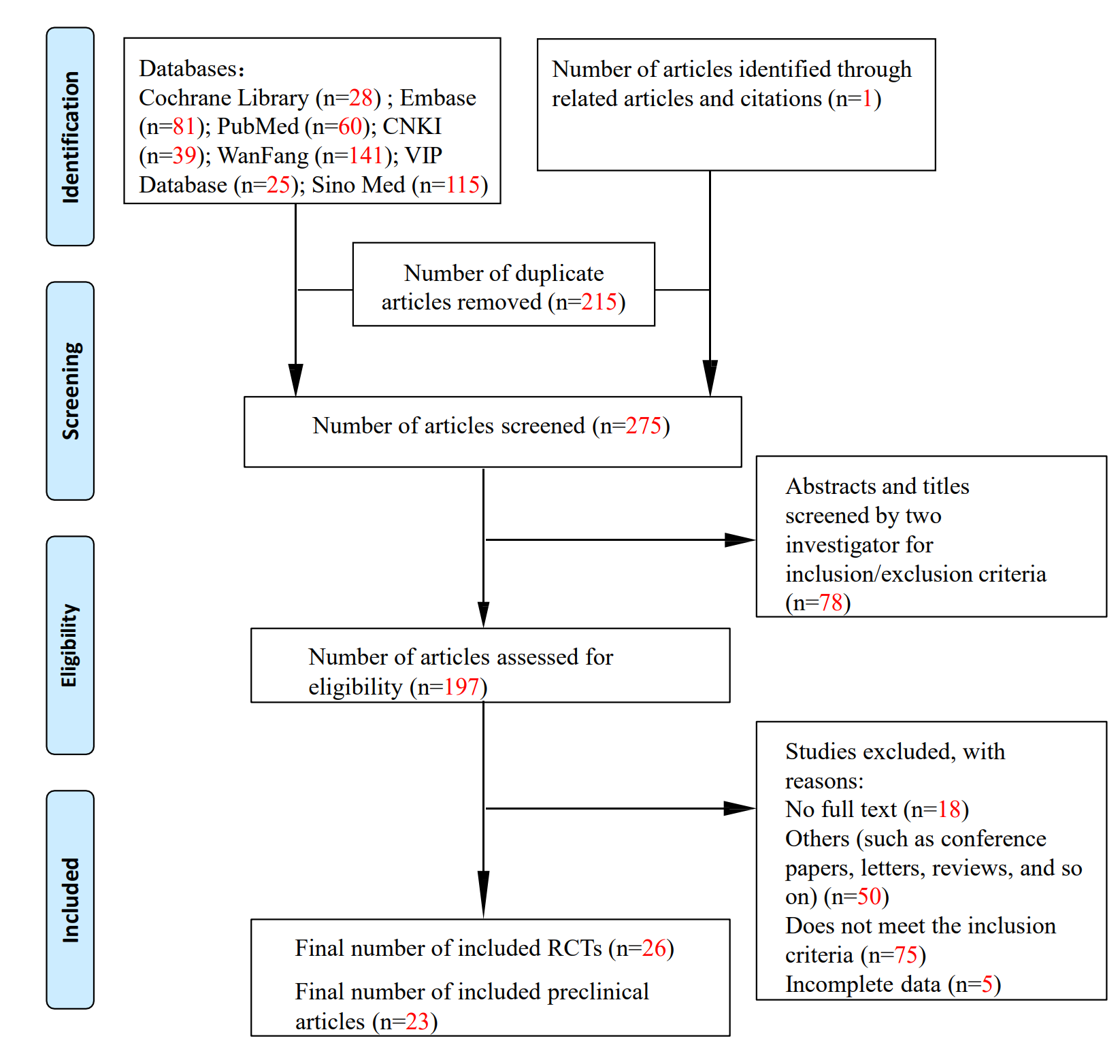

Supplement: Supplemental Material [file IANN_A_2329261_SM4652.zip › Supplementary Material Figure S1.tif]

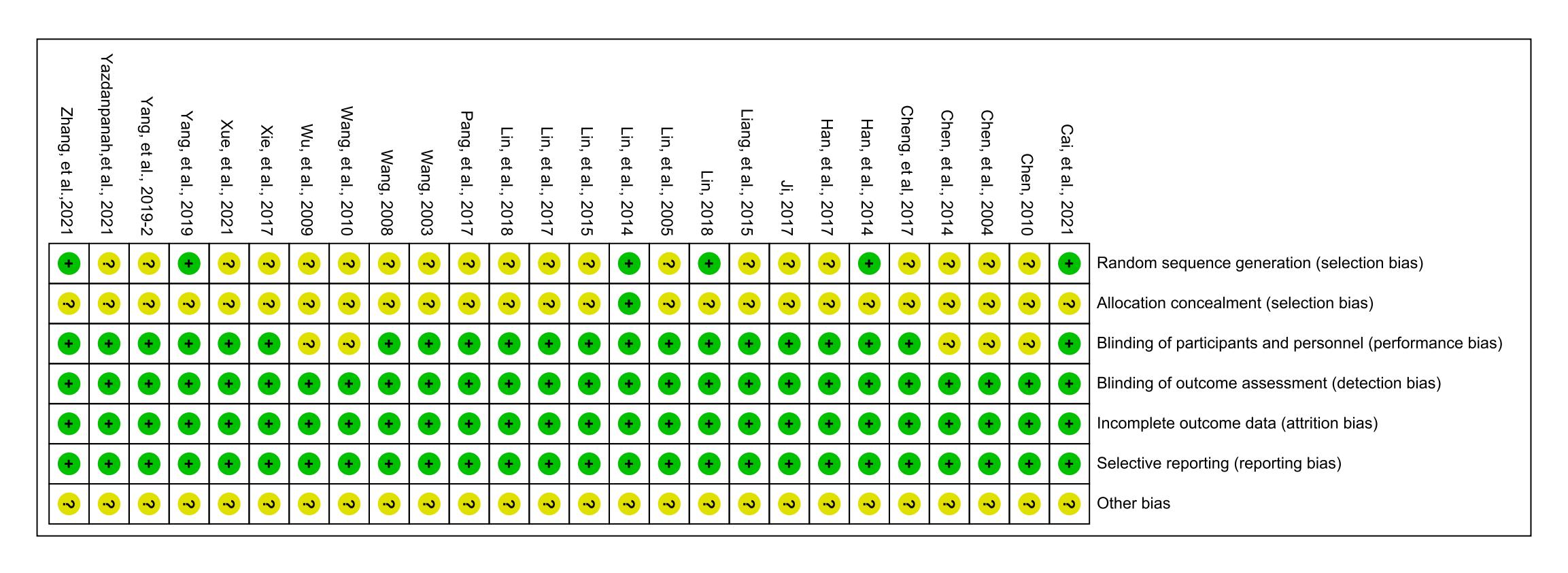

Supplement: Supplemental Material [file IANN_A_2329261_SM4652.zip › Supplementary Material Figure S2.jpg]

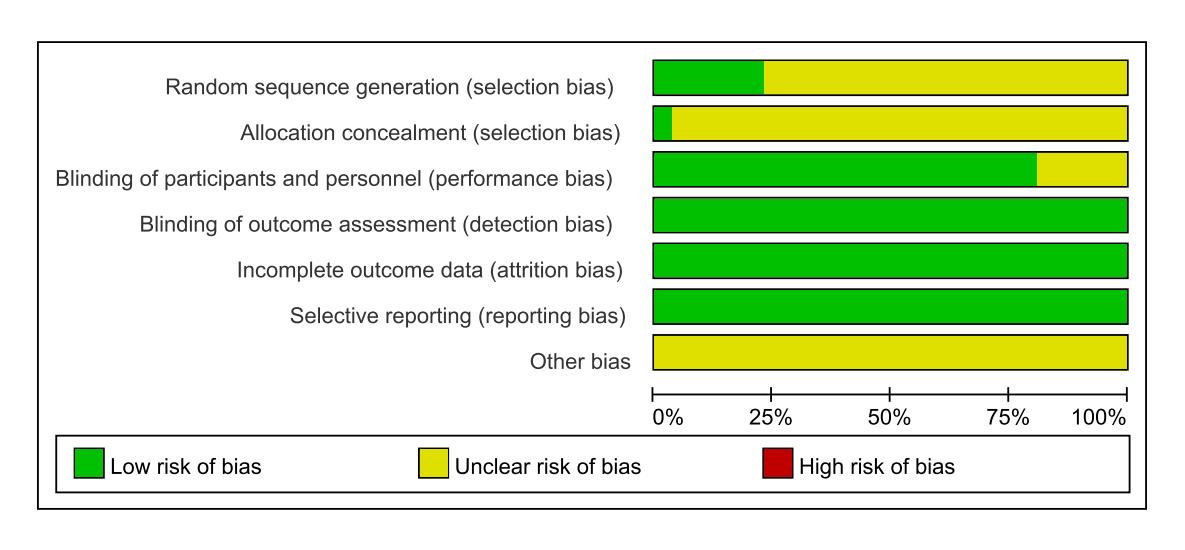

Supplement: Supplemental Material [file IANN_A_2329261_SM4652.zip › Supplementary Material Figure S3.jpg]

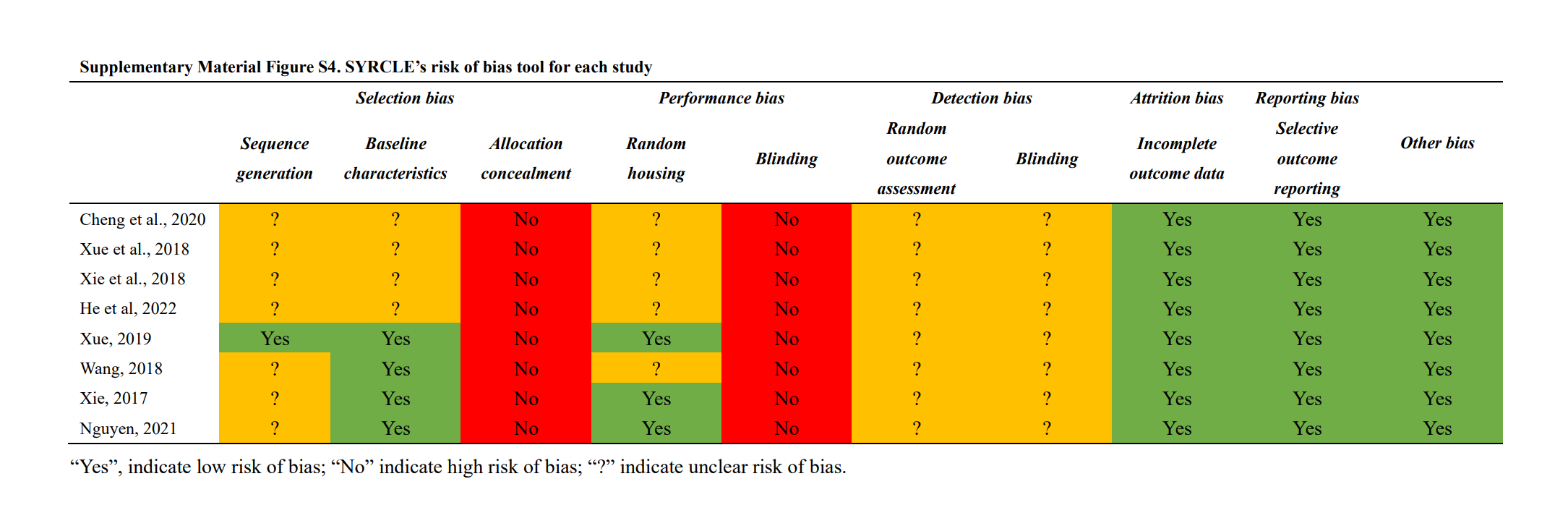

Supplement: Supplemental Material [file IANN_A_2329261_SM4652.zip › Supplementary Material Figure S4.jpg]

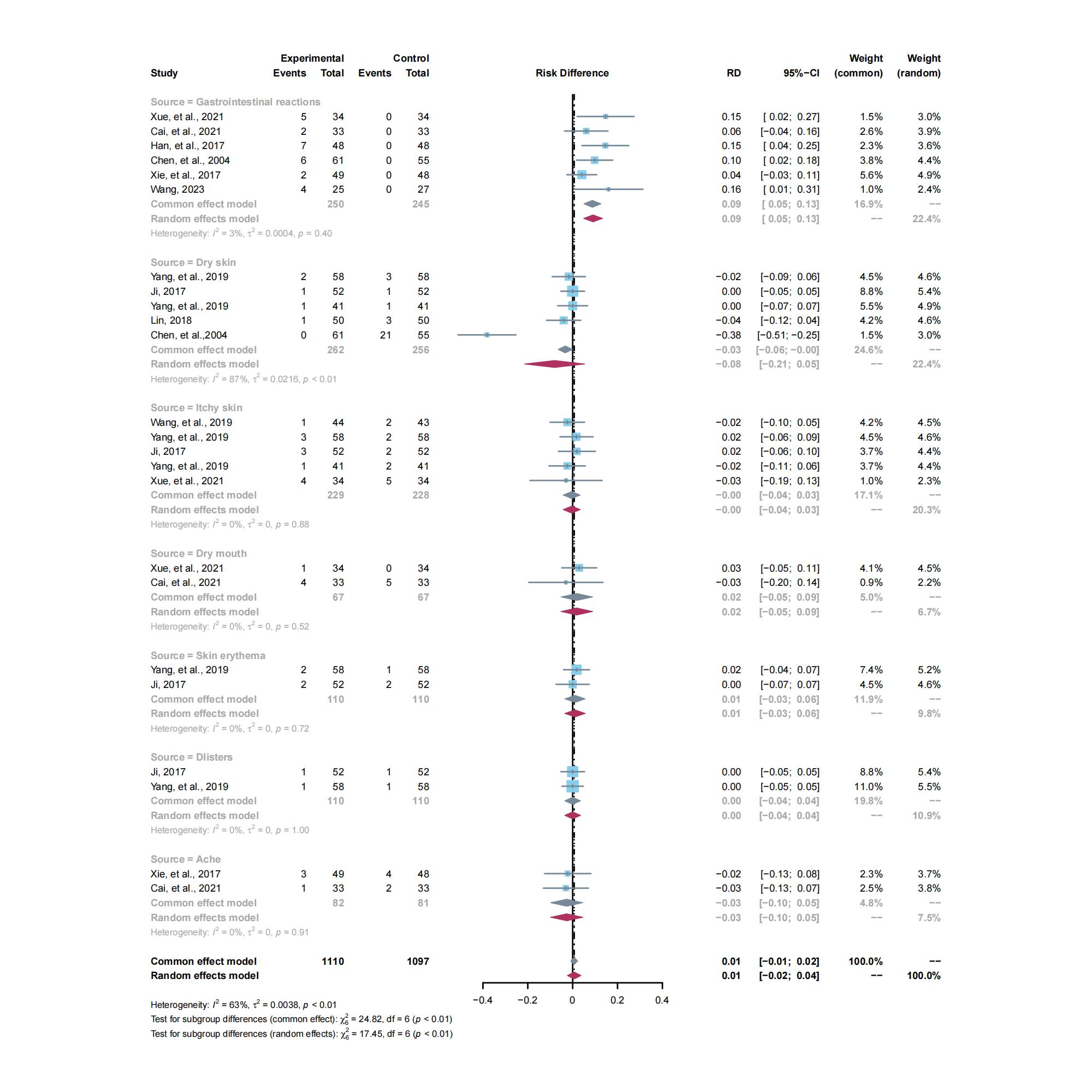

Supplement: Supplemental Material [file IANN_A_2329261_SM4652.zip › Supplementary Material Figure S5.jpg]

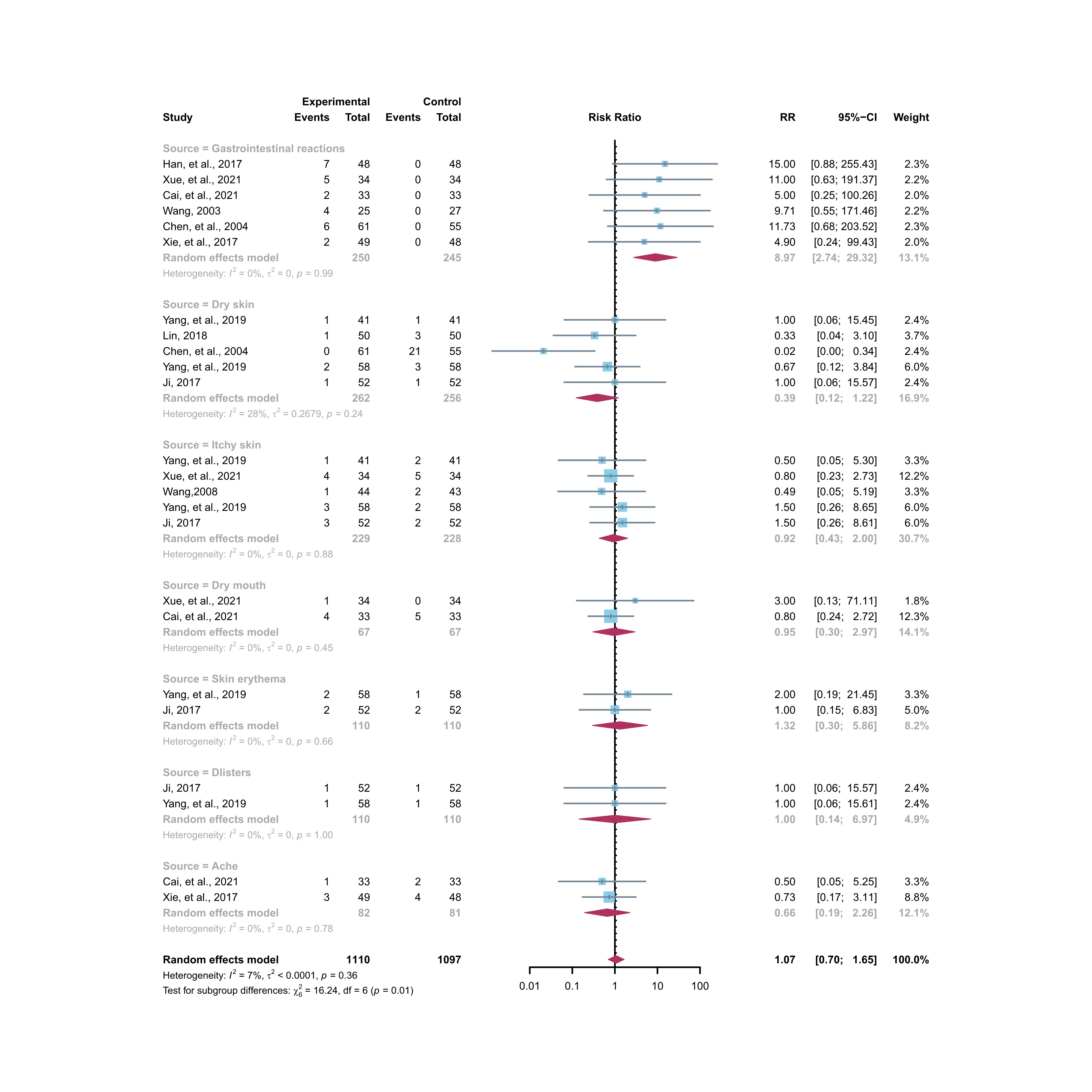

Supplement: Supplemental Material [file IANN_A_2329261_SM4652.zip › Supplementary Material Figure S6.jpg]
